# A combined microRNA and transcriptome analyses illuminates the resistance response of rice against brown planthopper

**DOI:** 10.1186/s12864-020-6556-6

**Published:** 2020-02-10

**Authors:** Jiaoyan Tan, Yan Wu, Jianping Guo, Huimin Li, Lili Zhu, Rongzhi Chen, Guangcun He, Bo Du

**Affiliations:** 1 State Key Laboratory of Hybrid Rice, College of Life Sciences, Wuhan University, Wuhan, 430072 China; 2 Institute for Biosciences and Biotechnology Research, University of Maryland, College Park, MD 20850 USA

**Keywords:** Brown planthopper, *BPH6*, Integrated analysis, miRNA-mRNA interaction, Resistance mechanism

## Abstract

**Background:**

The brown planthopper (BPH, *Nilaparvata lugens* Stål) is a kind of phloem-feeding pest that adversely affects rice yield. Recently, the BPH-resistance gene, *BPH6*, was cloned and applied in rice breeding to effectively control BPH. However, the molecular mechanisms underlying *BPH6* are poorly understood.

**Results:**

Here, an integrated miRNA and mRNA expression profiling analysis was performed on BPH6-transgenic (BPH6G) and Nipponbare (wild type, WT) plants after BPH infestation, and a total of 217 differentially expressed miRNAs (DEMs) and 7874 differentially expressed mRNAs (DEGs) were identified. 29 miRNAs, including members of miR160, miR166 and miR169 family were opposite expressed during early or late feeding stages between the two varieties, whilst 9 miRNAs were specifically expressed in BPH6G plants, suggesting involvement of these miRNAs in BPH6-mediated resistance to BPH. In the transcriptome analysis, 949 DEGs were opposite expressed during early or late feeding stages of the two genotypes, which were enriched in metabolic processes, cellular development, cell wall organization, cellular component movement and hormone transport, and certain primary and secondary metabolite synthesis. 24 genes were further selected as candidates for BPH resistance. Integrated analysis of the DEMs and DEGs showed that 34 miRNAs corresponding to 42 target genes were candidate miRNA-mRNA pairs for BPH resistance, 18 pairs were verified by qRT-PCR, and two pairs were confirmed by in vivo analysis.

**Conclusions:**

For the first time, we reported integrated small RNA and transcriptome sequencing to illustrate resistance mechanisms against BPH in rice. Our results provide a valuable resource to ascertain changes in BPH-induced miRNA and mRNA expression profiles and enable to comprehend plant-insect interactions and find a way for efficient insect control.

## Background

Rice is a primary food in China and other Asian countries (Normile 2008). The brown planthopper (BPH) is one of the most harmful insect pests of rice, which in modern rice cultivation causes severe damage and lead to large annual economic losses [1, 2]. As a typical vascular-feeding insect, BPH sucks the phloem sap of rice and results in extensive dwarfing, wilting, browning and drying of the plants. Furthermore, BPH serves as a vector to transmit viral disease [1, 2]. In the cultivation practice, BPH has developed resistance to most insecticides. The most economic and environment-friendly option for BPH control is to grow resistant rice varieties.

Since the report of the first BPH-resistance gene, *BPH1* in 1969 [3], more than 30 ones have been identified and mapped from wild and cultivated rice germplasms [2]. 12 of them, *BPH14*, *BPH26/2*, *BPH3*, *BPH29*, *BPH32*, *BPH18* and *BPH9/1/7/10/21* were characterized by map-based cloning approaches [4–10]. The structure and localization of BPH-resistance proteins provides a model system for determining the molecular basis of rice-BPH interaction. *BPH14* encodes an NLR (nucleotide-binding and leucine-rich repeat) protein that localizes to the cytoplasm and nucleus [4], *BPH1/2/7/9/10/18/21/26* encode NLR proteins that localize to the endomembrane [10], *BPH3* encodes a lectin receptor kinase that localize to the plasma membrane [6] whilst *BPH29* encodes a nucleus-localized B3 domain-containing protein [7]. Recently, we cloned another BPH-resistance gene, *BPH6*, encoding a yet uncharacterized protein in the exocyst and interacts with OsEXO70E1, an exocyst subunit [11]. However, the *BPH6*-mediated molecular mechanisms against BPH remain largely undefined.

Transcript profiles contribute to our understanding of the defense mechanisms of rice against BPH. Previously, the transcriptional profiles of resistant cultivar B5 and susceptible cultivar MH63 were reported using a cDNA microarray. Expression of genes involved in an array of signaling pathways, oxidative stress, pathogen-related, and macromolecule degradation was evidently enhanced, whilst expression of those involved in the flavonoid pathway, photosynthesis and cell growth was reduced upon BPH infestation [12–14]. Recently, a microarray analysis of Rathu Heenati and TN1 under BPH infestation revealed that transcription factors and plant hormones played important roles in the defense response [15, 16]. RNA sequencing of the *BPH15* introgression line and recipient line before and after infestation by BPH identified chief defense mechanisms associated with transcription factors, hormone signaling pathway, and MAPK cascades [17].

MicroRNAs (miRNAs) are ~ 21-nucleotide-long regulatory RNAs produced from endonucleolytic processing of single-stranded hairpin precursors in animal and plant [18]. miRNAs specifically regulate target gene expression through binding complementary sequences to degrade mRNA or inhibit translation [19]. Plant miRNAs are involved in many development processes, including hormone signal transduction, and leaf, floral, shoot, root and vascular development [20–22], and play significant roles in abiotic and biotic stress responses [23–28]. miR160 is associated with local defense and systemic acquired resistance to potato late blight [24]. miR166, miR169 and miR319 participate in the regulation of rice immunity against the blast fungus *Magnaporthe oryzae* [25–28]. However, few miRNAs have been revealed functioning in insect response. BPH-responsive miRNAs were investigated from resistant rice in comparison with susceptible plants [29]. miR156 and miR396 negatively regulated BPH resistance through regulating Jasmonic acid (JA) and flavonoid biosynthetic pathways, respectively [30, 31].

Although BPH responsive transcriptomes profiling of miRNAs and mRNAs have been reported independently, integrated expression profiling of miRNAs and their target genes associated with the interaction of rice and BPH has not been studied. To further reveal the molecular mechanism of rice responding to BPH, high-throughput sequencing was applied to analyze the miRNA and mRNA expression profiles in BPH fed seedlings. Upon integration of these two datasets, a total of 38 miRNAs, 24 genes and 34 miRNAs corresponding to 42 target genes were identified. Our result is a valuable resource for genome-wide studies on BPH responsive genes, and the resistance mechanisms mediated by miRNAs in rice.

## Results

### Evaluation of BPH resistance

**Fig. 1:**
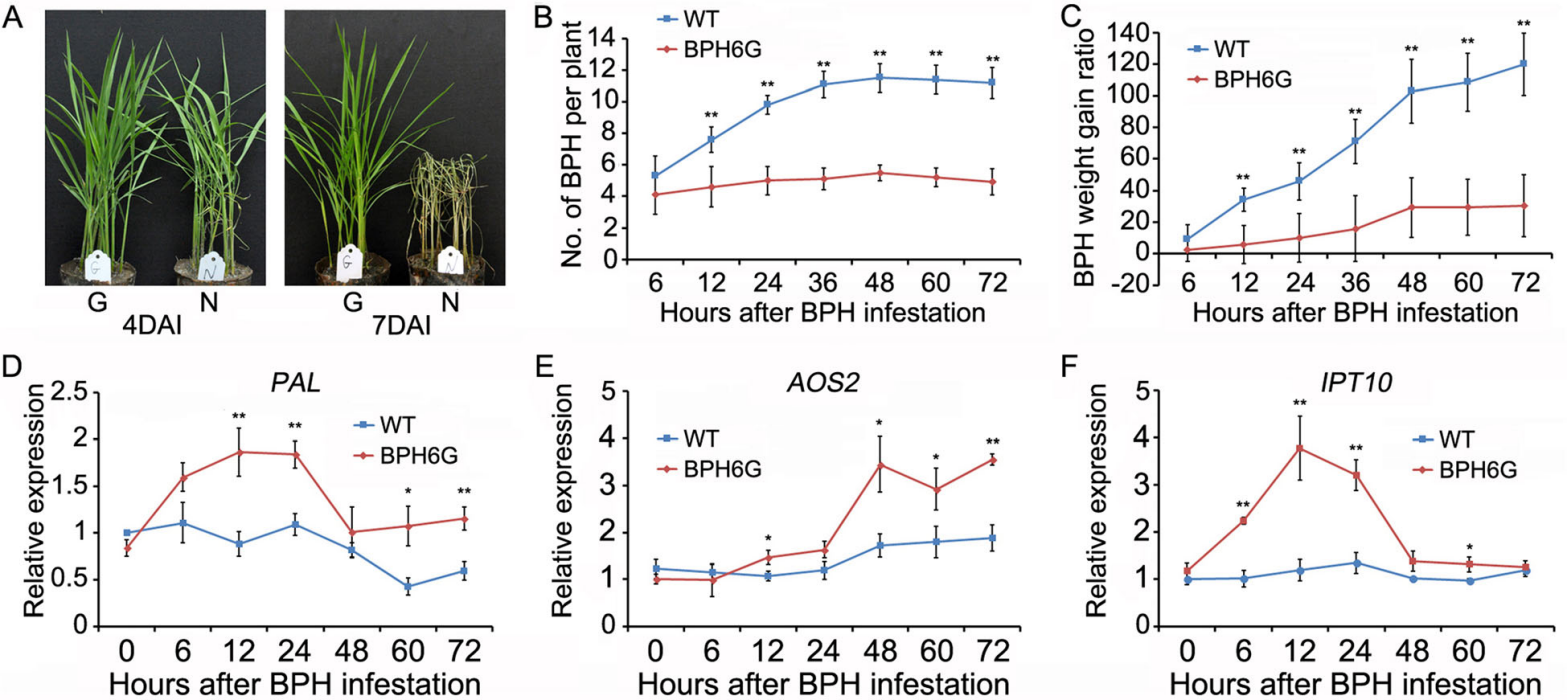
BPH resistance evaluation of the BPH6G and WT plants. **a**, Image of the BPH resistance evaluation of the BPH6G and WT seedlings. G stands for BPH6G, N stands for WT; DAI, days after infestation. **b**, Settling of BPH nymphs on the BPH6G and WT plants in the host choice test. **c**, BPH weight gain ratio on the BPH6G and WT plants. **d-f**, Expression of phytohormone synthesis-related genes in the BPH6G and WT plants after BPH infestation. Rice *TBP* was used as a reference control. Genes expression was quantified relative to values obtained from non-infested WT. Data represent means ± SD of ten independent experiments (**b-c**) and three independent biological repeats (**d-f**). Asterisks indicate significant differences revealed by one-way ANOVA at *P* < 0.05 (*) and *P* < 0.01 (**), respectively

In this study, a genomic fragment containing *BPH6* with its native promoter was transferred into the BPH susceptible wild type (WT), *Oryza sativa subsp. japonica* cv. Nipponbare, and got *BPH6*-transgenic plants (BPH6G). The homozygous T_2_ transgenic lines were analyzed for BPH resistance using the bulk seedling test. WT plants began to wither on the 4th day and died on the 7th day after BPH infestation, but the BPH6G plants were still alive (Fig. 1a). In the BPH host choice test, the average number of BPHs settled on WT increased rapidly from 6 to 48 h, whereas those on the BPH6G lines remained relatively constant over 72 h (Fig. 1b). Moreover, the ratio of weight gain was significantly less for BPH fed on the BPH6G plants than those on WT from 12 to 72 h (*P* < 0.01 at 12 h) (Fig. 1c).

In our previous work, the levels of salicylic acid, JA-Ile and cis-zeatin were induced to high levels from 3 to 24 h after BPH infestation in BPH6G compared to WT [11]. Phytohormone synthesis-related genes, *PAL* (phenylalanine ammonia-lyase), *AOS2* (allene oxide synthase 2) and *IPT10* (isopentenyl-transferase 10) were selected for expression analysis in BPH6G and WT plants after BPH infestation. Expression of *PAL* and *IPT10* increased more rapidly in the BPH6G plants from 6 to 24 h, whilst the expression levels of *AOS2* increased after 48 h in both plants (Fig. 1d-f). RNA was isolated from the leaf sheathes of the BPH6G and WT plants from 0 to 72 h after BPH feeding, and divided into non-infested controls (0 h), early feeding stages (including 6, 12 and 24 h), and late feeding stages (including 48, 60 and 72 h) for high-throughput sequencing analysis.

### Small RNA library construction and sequencing

Total reads of 9,034,925 to 14,016,694 were got in S0, S_early, S_late, R0, R_early and R_late libraries, respectively (Additional file 1: Table S1). After removing all low-quality reads, poly A, incorrect adaptors and reads of < 20 nt and > 24 nt, a total of 4,503,508 to 8,547,717 clean reads remained in the 18 libraries, respectively (Additional file 1: Table S1). In the 18 libraries, the main size classes were 21 nt and 24 nt followed by 22 nt and 23 nt as previously reported for rice small RNAs (Additional file 2: Fig. S1A). Approximately 20.16–23.09% (S0), 23.68–26.35% (S_early), 19.88–24.39% (S_late), 30.66–31.04% (R0), 31.37–35.58% (R_early) and 23.57–23.67% (R_late) of the clean reads were assigned to the miRBase database (Additional file 1: Table S1). Rice miRNA is the most thoroughly studied monocot miRNA, and 738 mature miRNAs were identified in the miRbase (release 22). Accordingly, we analyzed the 738 known miRNAs in our data.

### DEMs in the BPH6G and WT plants before and after BPH feeding

**Fig. 2:**
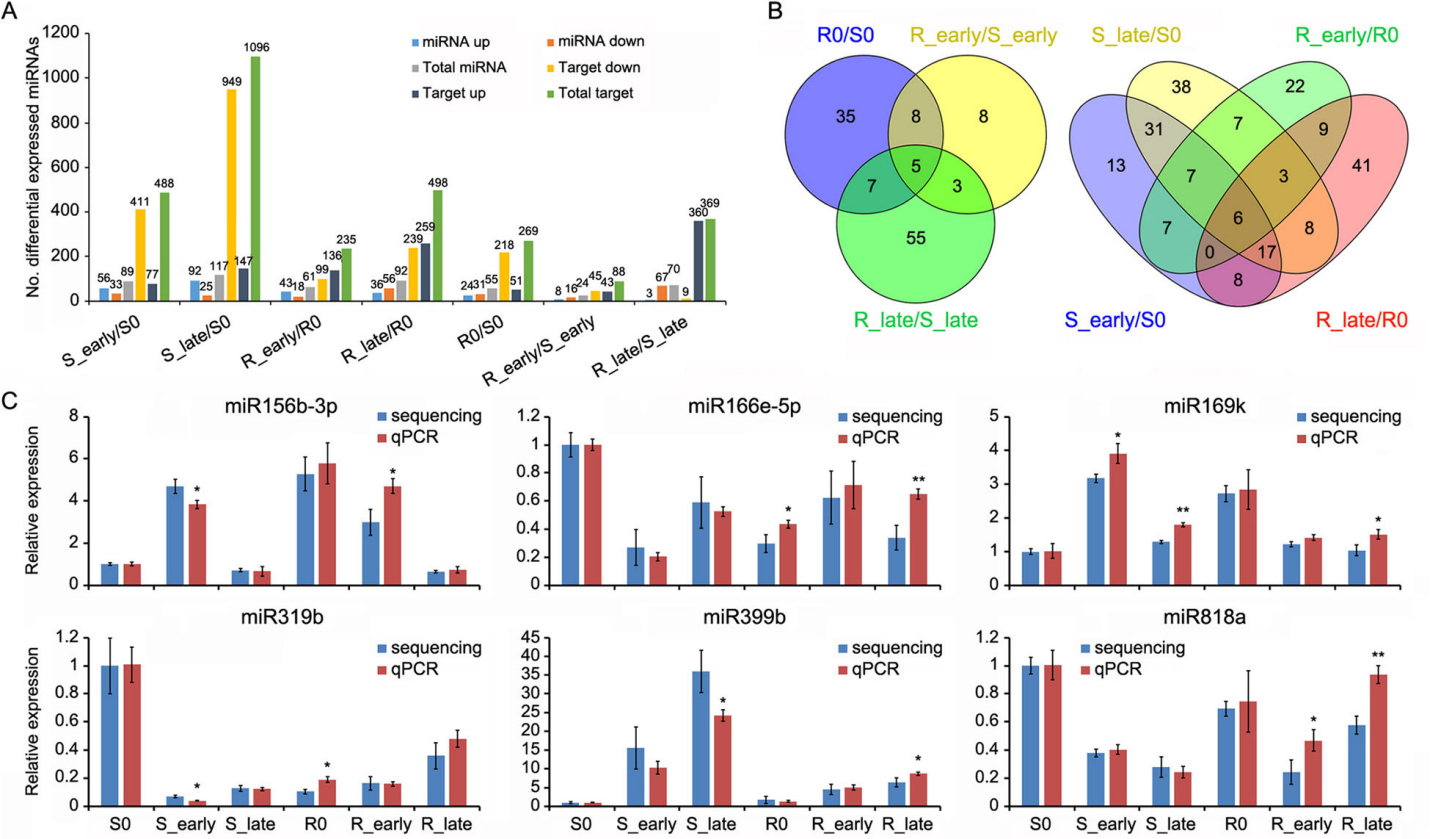
Differentially expressed miRNAs in the comparisons. **a**, Number of miRNAs and target genes up- or down-regulated in all comparisons (fold change > 2, *P* < 0.05). **b**, Venn diagrams of the unique and shared DEMs. **c**, Stem-loop RT-PCR to verify miRNA expression in the BPH6G and WT plants. miRNA expression was normalized by U6. Data represent the mean ± SD of three independent biological repeats. Asterisks indicate significant differences revealed by one-way ANOVA at *P* < 0.05 (*) and *P* < 0.01 (**), respectively

After normalization of the raw sequence reads, the average normalized reads of three independent biological replicates in the libraries were chosen for further analysis. The expression levels of miRNAs were compared amongst the different groups. Using fold changes ≥2, *P* < 0.05 of the average value of three replicates, 231 DEMs were detected, including 119 DEMs between the different varieties and 217 DEMs between different feeding stages (Fig. 2a-b). In the early feeding stages, there were more DEMs in WT (89) than in the BPH6G plants (61) (Fig. 2a). In the late feeding stages, the number of up-regulated DEMs (92) were four folds higher than down-regulated ones in WT (Fig. 2a), indicating that serious damage was caused by BPH.

To verify the data in miRNA sequencing, six DEMs were selected for quantitative stem-loop RT-PCR assays [32]. The results were broadly consistent to those from sequencing analysis, although expression of some miRNAs differed a little (Fig. 2c).

### Identification of miRNAs related to BPH resistance

miRNA expression differences in BPH resistant and susceptible rice before BPH attack was first compared. There were 55 DEMs, including 24 up-regulated and 31 down-regulated miRNAs in R0/S0 (Fig. 2a), many of which belonged to known miRNA families including miR156, miRNA160, miR166, miR169, miR1846, miR1861 and miR319 (Additional file 3: Table S2). Members of the miRNA families were reported to be involved not only in growth, development, grain size and hormone signaling, but also in response to biotic and abiotic stress [21–31]. These *BPH6* responsive DEMs might be involved in response to BPH.

**Fig. 3:**
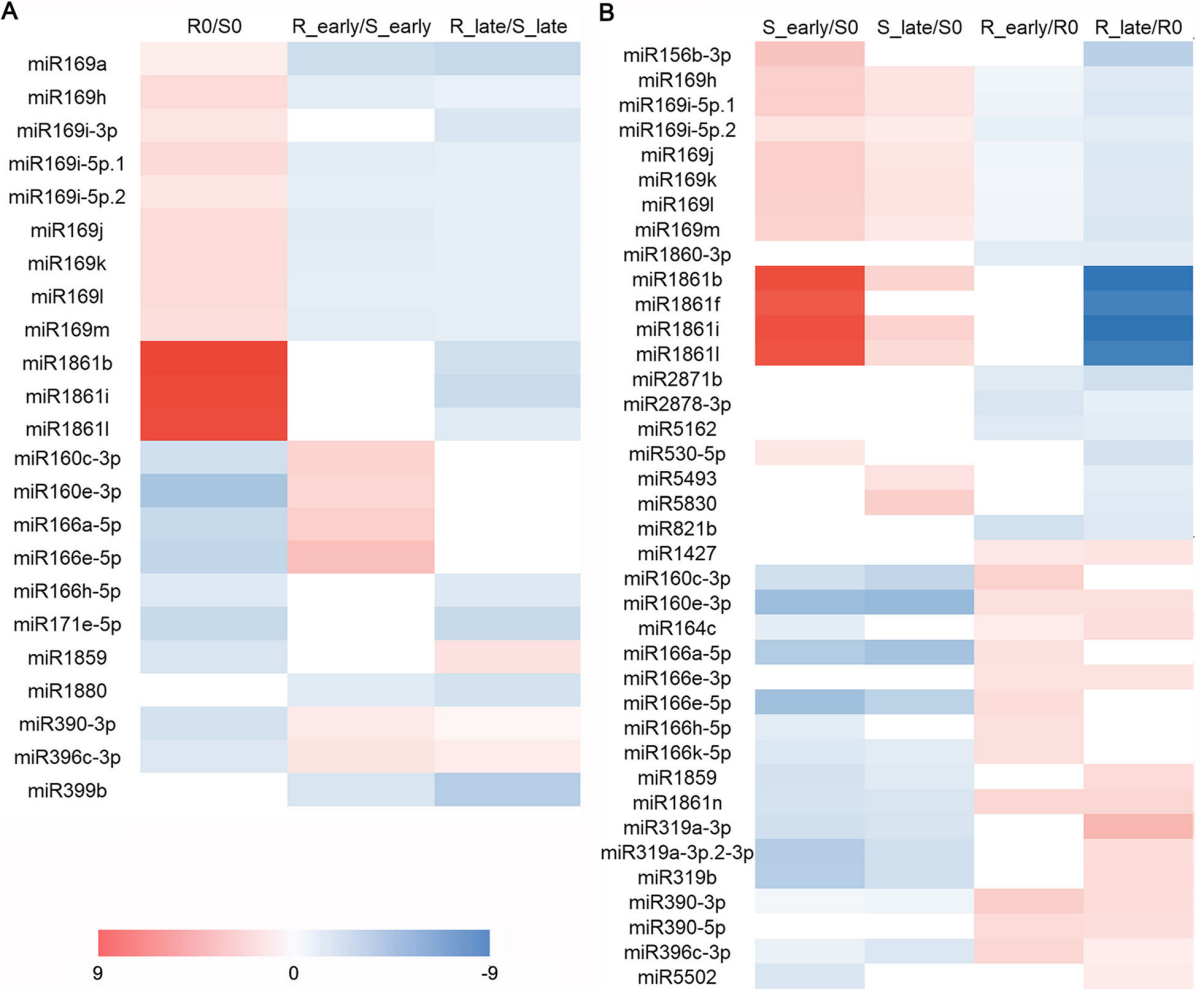
Potential BPH resistance-related miRNAs. **a**, Overlapping DEMs appeared in both varieties before and after BPH feeding. **b**, Overlapping DEMs appeared at the early and late feeding stages of both varieties and specially DEMs in the BPH6G plants. Colors represent fold-change values

To identify miRNAs related to plant resistance responses, Venn diagrams were used to show the DEMs appeared in the BPH6G plants compared to WT (R0/S0, R_early/S_early and R_late/S_late) (Fig. 2b). There were 23 overlapping DEMs in the comparisons (Fig. 2b), 18 of which showed opposite expression before and after BPH feeding (Fig. 3a, Additional file 3: Table S2). Members of the miR169 family were up-regulated before BPH feeding (R0/S0) and down-regulated after BPH feeding (R_early/S_early or R_late/S_late). In contrast, members of miR160 and miR166 families were down-regulated in R0/S0 and up-regulated in R_early/S_early or R_late/S_late.

The DEMs in early and late feeding stages of the two varieties (S_early/S0, S_late/S0, R_early/R0 and R_late/R0) were analyzed by Venn diagrams (Fig. 2B). A total of 63 DEMs were expressed in R_early/R0 or R_late/R0 and 9 specifically expressed in both R_early/R0 and R_late/R0 (Fig. 2b). Furthermore, 29 DEMs were opposite expressed in BPH6G and WT plants after BPH feeding (Fig. 3b, Additional file 4: Table S3). Among them, members of the miR169 family, miR156b-3p and miR396c-5p were down-regulated, whilst members of the miR160 and miR166 families were up-regulated in *BPH6*-trangenic plants. In addition, members of miR1861 and miR319, and other miRNAs appeared opposite expression in BPH6G and WT plants after BPH feeding, or were specifically expressed in both R_early/R0 and R_late/R0 (Fig. 3b).

### General mRNA expression profiles

mRNA libraries were constructed to analyze gene expression and to profile all miRNA targets that were differentially expressed in response to BPH feeding. Total reads of 95,471,364 to 111,697,630 were sequenced from 18 mRNA libraries. After deletion of low-quality reads in samples from the BPH6G plants, 84.70–89.99% of the reads were mapped to 28,988–30,383 rice genes (Additional file 5: Table S4). In the replicates from WT, 82.67–90.84% of the reads were mapped to 28,838–30,006 rice genes (Additional file 5: Table S4).

**Fig. 4:**
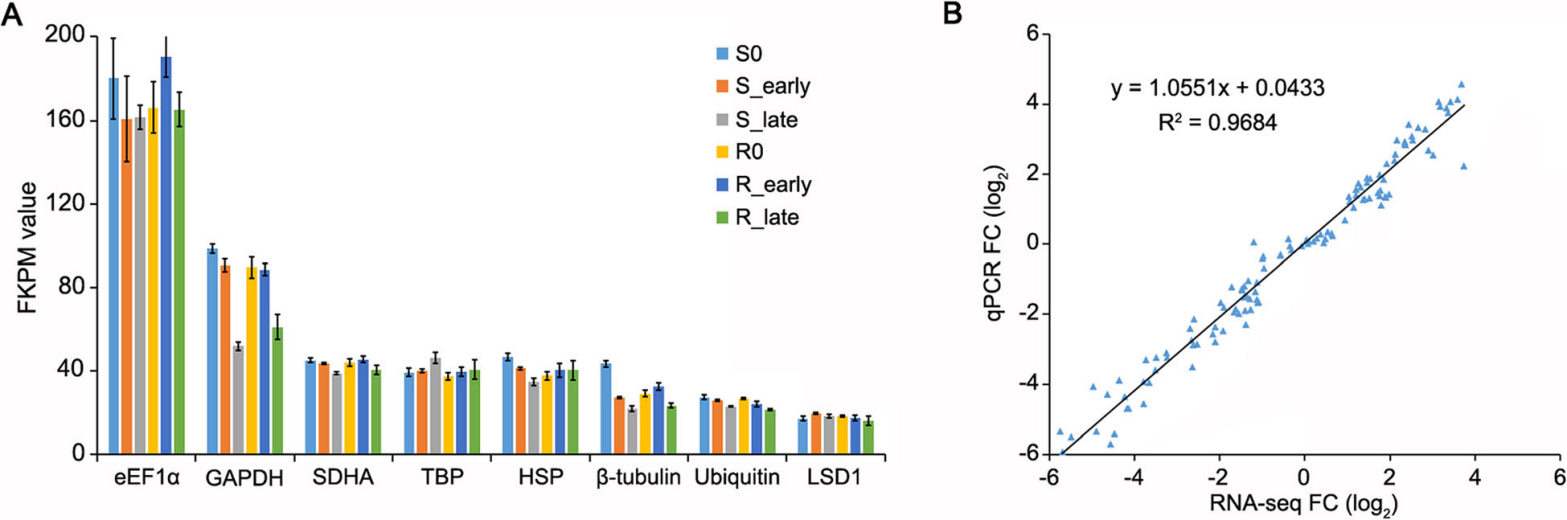
Expression profiles of mRNAs. **a**, FKPM values of *eEF1α*, *GAPDH*, *SDHA*, *TBP*, *HSP*, *β-tubulin*, *Ubiquitin* and *LSD1* from RNA-sequencing data. **b**, Pearson correlation scatter plots of comparisons of gene expression fold-changes measured by sequencing and qRT-PCR. Rice *TBP* was used as an internal reference. Gene expression was quantified relative to values obtained from non-infested samples. Data represent means of three independent biological repeats. Both x and y-axes are shown in the log2 scale. Pearson’s correlation coefficient is indicated by R

Considering that some reference genes are suppressed in host-herbivore interaction [33], we carefully selected reference genes with stable expression during BPH infestation for qRT-PCR analysis. Eight frequently used reference genes, *eEF1α* (*Os03g08020*), *GAPDH* (*Os02g38920*), *SDHA* (*Os07g0424*), *TBP* (*Os03g45410*), *HSP* (*Os03g31300*), *β-tubulin* (*Os03g56810*), *Ubiquitin* (*Os03g03920*) and *LSD1* (*Os12g41700*) were selected to evaluate the respective FPKM values extracted from our RNA-seq data (Fig. 4a). *eEF1α*, *GAPDH* and *β-tubulin* were significantly reduced in S_late and R_late, and *LSD1* was stable but relatively low. Combined with our previous results [11, 33], *TBP* was used as the reference gene for qRT-PCR analysis.

### DEGs in the BPH6G and WT plants before and after BPH feeding

There were 8577 DEGs (log_2_FC ≥ 1, FDR < 0.05) detected in this, including 4608 between the different varieties and 7874 between different feeding stages (Table 1). DEGs in the BPH6G and WT plants at different feeding stages were hierarchically clustered. Amongst the four comparisons, the expression patterns of the DEGs were similar, showing consistent up- or down-regulation (Additional file 6: Fig. S2).

**Table 1:** Differential expression genes between the *BPH6*-transgenic and Nipponbare plants in response to BPH feeding

Comparison	FC > 2		FC > 5		Total
	Up	Down	Up	Down	
S_early/S0	965	1952	184	365	2917
S_late/S0	2262	4132	683	2171	6394
R_early/R0	1851	657	590	94	2508
R_late/R0	1356	1569	344	382	2925
R0/S0	649	2678	149	1273	3327
R_early/S_early	591	642	173	310	1233
R_late/S_late	999	1007	255	381	2006

S: Nipponbare; R: the *BPH6*-transgenic plants; 0, non-infested; early: early feeding stage; late: late feeding stage

During early feeding stages, more DEGs were up-regulated in BPH6G plants (1851) compared to WT (965) (Table 1), and the numbers with FCs ≥ 5 were more in BPH6G plants (590) than in the WT (184). Up-regulated DEGs (1851) were three-fold more than down-regulated ones (657), and the number of up-regulated DEGs with FCs ≥ 5 (590) were six-fold more than down-regulated ones (94) in the BPH6G plants. During late feeding stages, the number of up-regulated DEGs (1356) were similar to that of down-regulated ones (1569) in BPH6G plants. However, during early feeding stages, the down-regulated DEGs (1952) were almost two-fold more than up-regulated ones (965) in WT, indicating the response to BPH-induced wounding and physiological stresses. During late feeding stages, the number of DEGs in WT dramatically increased from 2917 to 6394, and the number with FCs ≥ 5 increased remarkably from 549 to 2854, indicating more serious damage to rice plants caused by BPH.

To verify the RNA-seq data, 30 DEGs were selected for qRT-PCR analysis. The qRT-PCR results were consistent with RNA-seq data, since the genes displayed similar fold-changes with a correlation ratio of R^2^ = 0.968 (Fig. 4b).

### Identification of genes related to BPH resistance

**Fig. 5:**
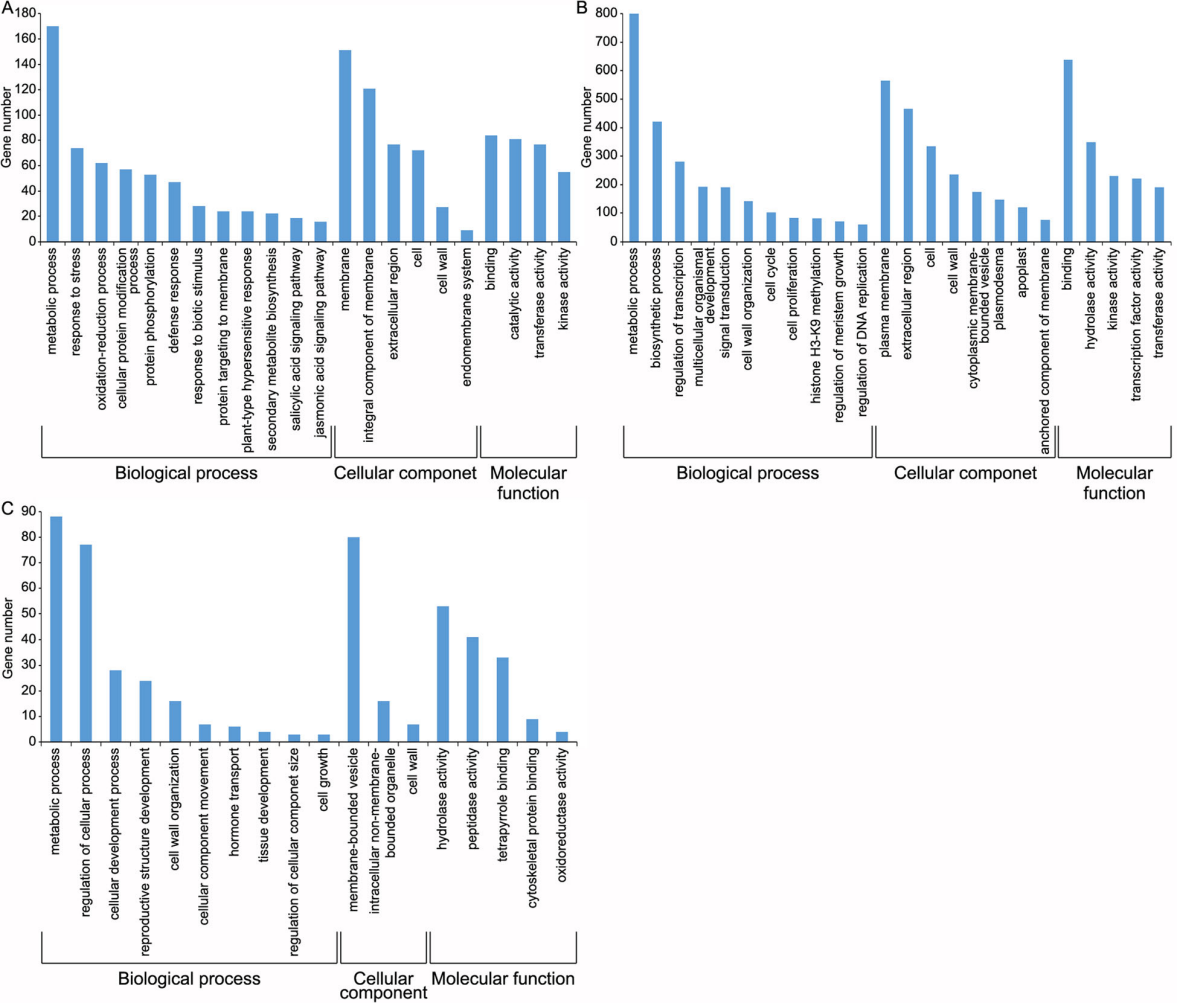
GO (Gene Ontology) analysis. Biological process, cellular component, and molecular function of up- (**a**) and down-regulated DEGs (**b**) in R0/S0, and the opposite expression DEGs (**c**) at the early or late feeding stages of the two varieties (*P* < 0.05). The x- and y-axis indicate the names of the clusters and the number of genes in a category, respectively

To investigate the function of *BPH6*, the sequencing data of BPH6G and WT plants before BPH feeding were compared. There were 3327 DEGs with FC ≥ 2, including 649 up-regulated and 2678 down-regulated ones (Table 1). These DEGs were analyzed by GO (gene ontology) enrichment to explores their functions. The up-regulated genes were enriched in defense, protein modification and protein targeting to membrane. Down-regulated genes were enriched in the regulation of transcription, signal transduction, cell wall organization and cell proliferation. In addition, these DEGs were enriched in plasma membrane, extracellular region, and cell wall for cellular component (Fig. 5a-b).

Next, Venn diagrams were used to analyze the possible BPH resistance-related genes in the DEGs of the two rice genotypes. In the BPH6G plants, there were 548 and 1572 DEGs down- and up-regulated respectively after BPH feeding; while in WT, 3127 and 1521 DEGs were respectively down- and up-regulated after BPH feeding (Additional file 7: Fig. S3A). To fully understand the function of these DEGs, GO enrichment analysis were performed. When the biological processes were considered, the up-regulated genes in the BPH6G plants and the down-regulated genes in WT were both enriched in cell wall organization or biogenesis, regulation of biological process, developmental growth, anatomical structure morphogenesis and single-multicellular organism process (Additional file 8: Fig. S4A, D). Down-regulated genes in the BPH6G plants and up-regulated genes in WT were both enriched in single-organism metabolic process, primary metabolic process and response to biotic stimulus and chemical (Additional file 8: Fig. S4B, C). Genes associated with hydrolase activity, Ras guanyl-nucleotide exchange factor activity and protein binding were most contrasting amongst the molecular function GO terms in the two rice varieties (Additional file 8: Fig. S4). Three cellular components of GO terms, external encapsulating structure, vesicle and intrinsic component of membrane were enriched, suggesting involvement of cell wall, vesicle and plant membrane in the response to BPH feeding (Additional file 8: Fig. S4).

To further streamline potential BPH resistance-related genes, the opposite expression DEGs during early and late feeding stages of two varieties were assessed. There were 949 DEGs in the BPH6G and WT plants after BPH feeding, of which, 935 were up-regulated in the BPH6G plants and down-regulated in WT, whilst 14 were down-regulated in the BPH6G plants and up-regulated in WT (Additional file 7: Fig. S3B). GO enrichment analysis indicated that these resistance-related genes were enriched in metabolic process, cellular development, cell wall organization, cellular component movement and hormone transport for biological process, and membrane-bounded vesicle and cell wall for cellular component (Fig. 5c). For further information regarding the molecular and biochemical responses of rice after BPH infestation, BPH responsive DEGs were combined with KEGG processes (Kyoto Encyclopedia of Genes and Genomes). At the *P* < 0.05, the BPH responsive DEGs were enriched in key pathways. The up-regulated DEGs were involved in primary and secondary metabolite processes, such as limonene and pinene degradation, starch and sucrose metabolism, stilbenoid, diarylheptanoid and gingerol biosynthesis, and brassinosteroid biosynthesis. In contrast, amino and nucleotide sugar metabolism and diterpenoid biosynthesis were remarkably enriched among the down-regulated genes (Additional file 9: Table S5). Finally, 24 genes were differentially expressed in both the BPH6G and WT plants after BPH feeding, and were considered BPH resistance-related genes (Table 2). Of these DEGs, 23 were dramatically up-regulated in the BPH6G plants and down-regulated in WT after BPH infestation. A single gene was down-regulated in the BPH6G plants and up-regulated in WT. Among them, two genes encoding germin-like proteins, two lipid transfer proteins, two cytochrome P450 family proteins and two Rop guanine nucleotide exchange factors played important roles against BPH. The majority of these genes were enriched in response to stress, transport, cell wall organization, Rho GTPase activity and oxidation-reduction process, and were enriched in the extracellular region, cell wall, membrane and nucleus (Table 2).

**Table 2:** Candidate BPH resistance-related genes exhibiting opposite expression in early and late feeding stages of two rice genotypes

AccID	Fold change (log2)				Description	GO term (BP)	GO term (CC)
	S_early/S0	S_late/S0	R_early/R0	R_late/R0			
LOC_Os03g44880	−3.0699	−7.6904	6.2795	4.4616	Putative germin-like protein 3–2	response to stress	extracellular region
LOC_Os08g35760	−2.9850	−6.8739	5.0342	4.1843	Germin-like protein 8–14	divalent metal ion transport	extracellular region
LOC_Os02g44320	−2.6067	−5.5841	3.5159	2.1718	14 kDa proline-rich protein DC2.15	lipid transport	extracellular region
LOC_Os10g40420	−1.7318	−3.7710	3.7780	2.4650	Plant lipid transfer protein	lipid transport	extracellular region
LOC_Os07g18750	−1.5582	−3.2059	3.0342	1.9405	Plant lipid transfer protein DIR1	lipid transport	extracellular region
LOC_Os05g10330	− 1.1491	−3.4066	2.5768	1.2144	Similar to Stem 28 kDa glycoprotein.	metabolic process	extracellular region
LOC_Os03g04530	−1.7801	−4.5678	3.1341	1.7710	Cytochrome P450 family protein	oxidation-reduction process	extracellular region
LOC_Os06g28000	−2.2380	−3.8361	2.6389	1.3979	Protein of unknown function DUF239		extracellular region
LOC_Os01g21034	−2.0392	−4.2691	3.2111	1.4877	Pectinesterase	cell wall organization	cell wall
LOC_Os11g03160	−1.0261	−2.5074	2.3742	1.2931	Glycosyl transferase, family 8 protein	cell wall organization	membrane
LOC_Os05g34320	−1.4953	−4.2403	3.1718	1.6017	Glycoside hydrolase	carbohydrate metabolic	cell wall
LOC_Os01g47780	−1.3583	−3.5887	3.3331	1.6020	Fasciclin-like arabinogalactan protein 11	plant-type secondary cell wall biogenesis	anchored component of membrane
LOC_Os08g34320	−1.5146	−4.1849	3.6937	2.0720	Protein of unknown function DUF566.		
LOC_Os05g38000	−1.0291	−3.5098	2.9374	1.8637	Rop guanine nucleotide exchange factor 7	positive regulation of Rho GTPase activity	plasma membrane
LOC_Os07g29780	−1.1778	−3.9431	2.6677	1.1438	Rop guanine nucleotide exchange factor 3	positive regulation of Rho GTPase activity	plasma membrane
LOC_Os09g17660	−1.8677	−4.6194	3.5913	1.9719	HSP20-like chaperone protein	response to stress	cell
LOC_Os01g55560	−1.0358	−3.1142	3.8714	2.1315	Probable protein ABIL5	anatomical structure morphogenesis	SCAR complex
LOC_Os08g14700	−1.9333	−2.4197	2.4781	1.5860	Glucan endo-1,3-beta-glucosidase 7	regulation of meristem growth	cell
LOC_Os07g37850	−1.5484	−5.5175	3.4314	1.2854	Similar to LLA-115		cell
LOC_Os01g05840	−1.1587	−4.1744	3.0301	1.1690	Short-chain dehydrogenase TIC 32	oxidation-reduction process	
LOC_Os08g33660	−1.4375	−3.6294	3.4132	1.9560	Transcription factor MYB106	anatomical structure morphogenesis	nucleus
LOC_Os07g01530	−1.2440	−3.3855	2.5365	1.0732	NB-ARC domain containing protein.	defense response	nucleus
LOC_Os02g45420	−1.2912	−1.7180	1.7674	1.3400	Ethylene response factors	response to stress	nucleus
LOC_Os01g72270	1.5876	3.9238	−2.0629	−1.4023	Cytochrome P450, family 94, CYP94D	oxidation-reduction process	

GO terms were selected as the term with the lowest *P* value

*BP* biological process, *CC* cellular component

### Integrated analysis of miRNA and mRNA expression profiles

In most cases, miRNAs negatively regulate target mRNA through translation repression or mRNA degradation [19]. To correlate the identified miRNAs with their target genes, the psRNA target tool was used to predict miRNA targets on mRNAs using the parameters fold changes ≥2, *P* < 0.05 [34]. There were 89, 117, 61 and 92 DEMs that significantly and negatively correlated with 488, 1096, 235 and 498 target mRNAs in S_early/S0, S_late/S0, R_early/R0 and R_late/R0, respectively (Fig. 2a). In addition, 55, 24 and 70 DEMs negatively correlated with 269, 88 and 369 target mRNAs in R0/S0, R_early/S_early and R_late/S_late, respectively (Fig. 2a).

To identify potential miRNA-mRNA pairs related to BPH resistance, 70 DEMs in R_early/R0 or R_late/R0 (Fig. 2b) and 29 DEMs at different feeding stages (Fig. 3b) were selected and negatively correlated with 656 target mRNAs (Additional file 10: Table S6). These miRNAs target different mRNAs during each feeding stage. For example, miR156b-3p was up-regulated in S_early/S0 and down-regulated in R_late/R0, which negatively correlated with 20 down-regulated target genes in S_early/S0 and 4 up-regulated ones in R_late/R0, respectively. However, the majority of these targets showed a similar trend of expression in the BPH6G and WT plants after BPH feeding (Additional file 10: Table S6). Excluding these miRNAs and their corresponding targets, 34 miRNAs corresponding to 42 target genes were differentially expressed in R_early/R0 or R_late/R0, or opposite expressed in the BPH6G and WT plants after BPH feeding, and selected as BPH resistance-related miRNA-mRNA candidates (Table 3).

**Table 3:** The miRNA-mRNA interactions related to plant resistance

AccID	Fold change (log_2_)				AccID	Fold change (log_2_)				Description
	S_early/S0	S_late/S0	R_early/R0	R_late/R0		S_early/S0	S_late/S0	R_early/R0	R_late/R0	
miR156b-3p	2.9038			−3.0044	LOC_Os02g40440	−2.4818	−4.8408	1.0794		GDSL-like lipase/acylhydrolase
					LOC_Os03g06940	−1.5343	−5.5008	1.8591		Beta-galactosidase
					LOC_Os07g05370		−0.8225	1.7330	1.2472	Probable receptor-like protein kinase
miR169h miR169i-5p.1 miR169j miR169k miR169l miR169m	2.3399 2.3485 2.3385 2.3553 2.2678 2.2304	1.3237 1.3876 1.2357 1.3497 1.3537 1.1487	− 0.6176 − 0.7214 − 0.6419 − 0.5419 − 0.5881 − 0.6025	−1.3974 − 1.5362 − 1.4945 − 1.4593 − 1.4666 − 1.6050						
					LOC_Os03g20450	−1.8218	−4.4633	2.3586	0.7232	Leucine Rich Repeat family protein, expressed.
					LOC_Os05g36990		−3.1973	2.9861	1.3373	Transcription repressor OFP13
					LOC_Os06g49390		−3.3648	1.3698		Disease resistance protein domain containing protein.
miR169i-5p.2	1.3897	1.0292	−0.9670	−1.2217	LOC_Os05g38980		−2.9599	1.5245		Putative respiratory burst oxidase homolog protein H
					LOC_Os07g46560	−1.2143	−5.6792	1.4218		E3 ubiquitin-protein ligase DIS1-like
					LOC_Os11g36180	−1.2127	−1.9053	2.1591		Leucine Rich Repeat family protein.
miR1861b miR1861f miR1861i miR1861l	8.7372 8.4214 8.1198 8.5830	2.1507 - 2.2432 1.8472		−8.8720 −8.1310 −8.9142 −8.2140						
					LOC_Os04g58840 LOC_Os04g56850 LOC_Os10g40730	−1.4457 − 1.1866 − 1.3179	−3.1338 −4.3536 −4.0628	1.0975 2.3633 1.7690		Peptidase aspartic, catalytic domain protein Auxin response factor. Beta-expansin EXPB4.
miR5830		2.3722		−1.3373	LOC_Os01g58550	−0.3622	−1.9871	3.5266	2.4752	Methyladenine glycosylase
					LOC_Os01g62900		−1.1347	2.0992	1.0369	Delta 1-pyrroline-5-carboxylate synthetase
					LOC_Os05g43820	−1.1581	−3.9153	1.8119		Small GTP-binding protein OsRac2.
miR169o				−1.3769	LOC_Os01g58550	−0.3622	−1.9871	3.5266	2.4752	Methyladenine glycosylase
miR1849				−1.5045	LOC_Os06g10170		−1.7348	2.1211	1.4939	Flavin-containing monooxygenase FMO family protein.
miR1860-3p			−1.1943	−1.2435	LOC_Os09g17660	−1.8677	−4.6194	3.5913	1.9719	HSP20-like chaperone protein.
miR2871b			−1.3463	−2.0454	LOC_Os02g52000			3.3851	3.2822	Similar to Phi-1 protein
					LOC_Os04g58870			1.3380	0.9474	Exocyst complex component EXO70A1
					LOC_Os12g10670	−1.4473	−4.5344	2.1135		AAA-type ATPase family protein
miR393b-3p				−1.0201	LOC_Os08g35760	−2.9850	−6.8739	5.0342	4.1843	Germin-like protein 8–14.
miR396c-5p				−1.0609	LOC_Os02g47470			1.8335	2.2926	Abscisic acid 8′-hydroxylase 1.
					LOC_Os03g21800			1.8898	1.4858	bZIP DNA-binding protein, Disease resistance
					LOC_Os03g47140		−1.2584	2.6187	1.7314	Growth-regulating factor 9.
miR397a				−1.1363	LOC_Os07g35480	0.4191		1.2744	1.3160	Glucan endo-1,3-beta-glucosidase 3
					LOC_Os09g27950	0.2413	0.3982	1.0214	1.0453	Beta-1,3-galactosyltransferase 7
					LOC_Os10g28240		0.3940	1.7770	1.2730	Calcium-transporting ATPase 8, plasma membrane-type.
miR530-3p			−0.9630	−2.4135	LOC_Os02g03280	0.8367	0.8573	1.5406	1.4134	Bax inhibitor-1 (BI-1) (OsBI-1).
miR5489				−3.6414	LOC_Os06g14490			1.4742	1.3802	Similar to Calmodulin-binding heat-shock protein.
miR5513			−1.0134	−0.7966	LOC_Os01g46870		−3.4105	4.2300		Similar to Ethylene-responsive transcription factor 5
					LOC_Os04g42860	−1.0897	−3.1407	1.7963		Lipase, GDSL domain containing protein.
					LOC_Os09g37270	−0.4175	−1.3617	1.7923	1.0559	Rop nucleotide exchanger, PRONE protein.
miR818a miR818b miR818e	−0.7157 − 0.6001 -	−0.9040 − 0.7481 − 0.9617	− 1.0855 − 1.1284 − 1.2987		LOC_Os03g08530		−2.7887	1.2552		Similar to Alanine aminotransferase.
					LOC_Os05g38480	−1.5722	−3.2472	1.2204		Kinesin, motor region domain containing protein.
					LOC_Os05g50260		−4.6151	1.6969		Similar to Polygalacturonase PG2.
miR169i-5p.2	1.3897	1.0292	−0.9670	−1.2217	LOC_Os07g31840	−1.0578	−3.2976	1.2127		Receptor-like protein 4.
miR1860-3p			−1.1943	−1.2435						
miR169g				−1.2426	LOC_Os11g10770	0.3442		1.1902	1.0504	Leucine-rich repeat, typical subtype containing protein.
miR169o				−1.3769						
miR2871a-3p				−1.5960						
miR395h miR395p miR395q				−2.3622 −1.4083 − 1.9536	LOC_Os05g07060		−2.0727	3.0620	1.4235	Fasciclin-like arabinogalactan protein 11
miR397a				−1.1363	LOC_Os03g03510	0.7319	0.5969	1.8286	1.7473	Similar to CBL-interacting protein kinase 9.
miR529b	0.7123	−0.8500		−1.6261						
miR5493		1.4197		−1.1295	LOC_Os06g48030			2.1537	2.2485	Peroxidase 16
miR5830		2.3722		−1.3373						
miR2878-3p			−1.6194	−1.0505	LOC_Os07g30690		−2.9111	2.5734		7-deoxyloganetic acid glucosyltransferase
miR818a	−0.7157	−0.9040	−1.0855							
miR818b	−0.6001	−0.7481	−1.1284							
miR818e		−0.9617	−1.2987							

**Fig. 6:**
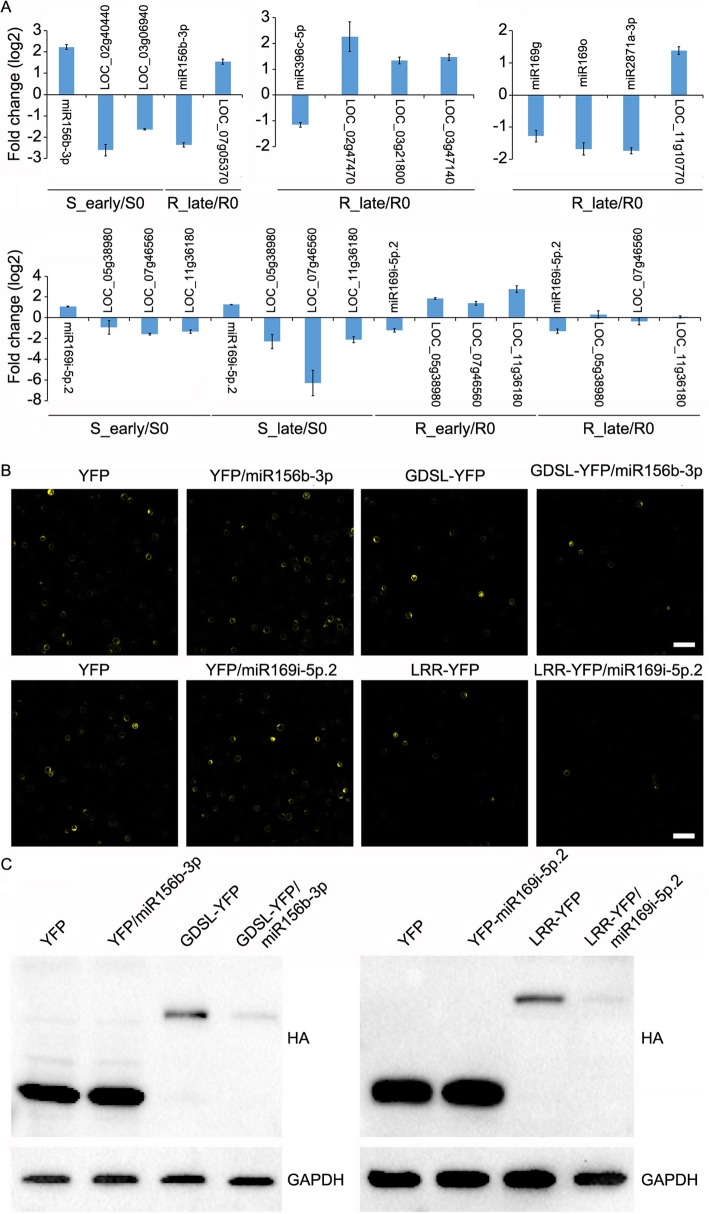
Negative regulation of miRNAs on their target genes. **a**, Contrasting expression patterns of miRNAs and their targets. Data represent the mean ± SD of three independent biological experiments. **b**, Fluorescence micrographs of rice protoplasts transfected with blank YFP plasmids, miRNAs and target gene plasmids. Scale bar, 50 μm. **c**, Western blot analysis of *YFP* and the target genes in rice protoplasts using anti-HA and anti-GAPDH antibodies

To validate whether these miRNAs negatively regulate target expression, four miRNAs and their targets were selected for qRT-PCR verification. The results indicated that miR156b-3p negatively regulated *LOC_Os02g40440* and *LOC_Os03g06940* in S_early/S0, and *LOC_Os07g05370* in R_late/R0, miR396c-5p negatively regulated *LOC_Os02g47470*, *LOC_Os03g21800* and *LOC_Os03g47140* in R_late/R0, and miR169g/o and miR2871a-3p negatively regulated *LOC_Os07g31840*. Furthermore, three target genes (*LOC_Os05g38980*, *LOC_Os07g46560* and *LOC_Os11g36180*) were down-regulated by miR169i-5p.2 in S_early/S0 and S_late/S0, up-regulated in R_early/R0, while unaffected in R_late/R0 (Fig. 6a).

miR156b-3p and miR169i-5p.2 with their targets encoding GDSL-like lipase (*GDSL*/*LOC_Os02g40440*) and Leucine Rich Repeat family protein (*LRR*/*LOC_Os11g36180*) respectively were selected for validation in rice protoplasts. Two plasmids of each pair, one encoding pri-miRNA, and the other YFP and HA fused target, were transfected into the protoplasts. In both cases, the inflorescence signal of the blank YFP plasmid could not be weakened by the pri-miRNAs, however, that of the targets could be significantly weakened by the respective pri-miRNAs (Fig. 6b). Western blot verified the results of the YFP signal at the protein level (Fig. 6c). These results indicate that miR156b-3p and miR169i-5p.2 down-regulate *GDSL* and *LRR* expression in rice cells, respectively.

## Discussion

Few studies have reported the use of combined miRNA and mRNA expression profiles to analyze the responses of herbivore insects in plants, excluding studies on aphid-induced miRNA expression [35]. This study was the first to report the combined analysis of miRNA and mRNA expression profiles in BPH-infested rice, enhancing our understanding of the regulatory mechanisms of miRNA-mRNA in rice after BPH attack.

In this study, the average number and the weight gain rate of BPHs increased rapidly from 6 to 48 h and remained gently after 48 h on WT (Fig. 1b-c). In addition, there were significant differences in the expression of hormone-related genes before and after 48 h in the BPH6G plants (Fig. 1d-f). These results demonstrate that the defense establishment and significant progression of BPH6G plants exists up to 48 h after BPH infestation. Therefore, RNA was divided into three groups, non-infested, early feeding stage (before 48 h) and late feeding stage (after 48 h).

Through the comparison of miRNA expression of the BPH6G and WT plants before and after BPH attack, a total of 217 known DEMs were identified (Fig. 2a-b). To identify miRNAs related to BPH response, the DEMs amongst seven comparisons: R0/S0, R_early/S_early, R_late/S_late, S_early/S0, S_late/S0, R_early/R0 and R_late/R0 were analyzed using Venn diagrams (Fig. 2b). 18 DEMs appeared in two of the comparisons (R_early/R0 and R_late/R0), whilst 61 DEMs appeared in both S_early/S0 and S_late/S0 (Fig. 2b), suggesting a lower number of miRNAs were involved in BPH defense responses in the BPH6G plants. 18 miRNAs were opposite expressed before and after BPH feeding in comparison to R0/S0, R_early/S_early and R_late/S_late, and 29 miRNAs were opposite expressed in the BPH6G and WT plants after BPH feeding (Fig. 3). Seventeen of the miRNAs, miR160c-3p/e-3p, miR166a-5p/e-5p/h-5p, miR169h/i-5p.1/i-5p.2/j/k/l, miR1859, miR1861b/j/l, miR390-3p and miR396c-3p, were present in both groups, suggesting their involvement in the defense response of rice against BPH are consistent with their respective roles in pathogen defense [24–26]. Previously, the BPH-responsive miRNAs were identified in a *BPH15* introgression line [29]. In both the *BPH15* introgression and the BPH6G lines, some miRNA exhibited similar expression trends, such as miR156b-3p, miR169h/i-3p/i-5p.1/i-5p.2/j/k/l/m/o, miR396c-5p, miR399j, miR530-5p and miR5513, suggesting a conserved and diverse resistance mechanisms against BPH mediated by *BPH6* and *BPH15*.

miR156, miR160, miR166, miR169 and miR396 were reported to participate in rice immune response against pathogens and insects. miR160 positively regulated potato defense to late blight [24]. miR166 positively regulated rice immunity against the blast fungus via post-transcriptional control of *EIN2* [26]. miR169, miR156 and miR396 negatively regulated rice immunity against the pathogens and BPH, respectively [25, 30, 31]. In the BPH6G plants, members of miR160 and miR166 family were upregulated, whilst those of miR156, miR396 and miR169 families were downregulated (Fig. 3b), implying their involvement in BPH response. In addition, miR1859 showed higher expression during heat stress treatment [22]. Members of the miR1861 family regulate starch accumulation and yield in rice [21], whilst miR390-TAS3-ARFs forms an auxin-responsive regulatory network controlling root growth [20]. These reports suggest that the miRNAs indirectly participate in BPH stress responses through altering the metabolic processes and hormone regulation. Interestingly, miR319 negatively regulated immunity to rice ragged stunt virus and blast fungus by repressing the expression of *OsTCP21*, leading to decrease JA-mediated defenses [27, 28], whilst members of the miR319 family were upregulated in the BPH6G plants (Fig. 3b), suggesting that miR319 might separately regulate rice immunity against BPH and pathogens.

To study BPH resistance related genes a wide range, RNA-sequencing analysis was performed in the BPH6G and WT plants under BPH infestation. Transcriptome analysis revealed notable differences in the response of the BPH6G and WT plants to BPH feeding. The inducible defense responses against BPH in BPH6G plants were more robust during early feeding stages compared with WT as a larger number of up-regulated DEGs (FCs ≥ 2) were detected in the BPH6G. In contrast, a larger number of DEGs were detected in WT during early and late feeding stages, indicating remarkable metabolic and physiological changes in WT after BPH feeding due to the absence of BPH resistance. In addition, up-regulated DEGs were much higher than down-regulated ones in the BPH6G plants, suggesting that the expression of genes associated with resistance in the BPH6G plants was up-regulated.

Previously, the transcript profiles of resistant rice cultivars revealed key defense mechanisms related to transcription factors, hormone signaling, MAPK cascades and pathogen-related genes. In this study, the DEGs were analyzed during early and late feeding stages in the two varieties to reveal the BPH6-mediated defense mechanisms. The GO enrichment analysis of the DEGs of BPH6G and WT plants before BPH feeding indicate that *BPH6* takes part in defense and stress, and other developmental and physiological process. There were 949 DEGs opposite expressed at early or late feeding stages between the two varieties, most of which were up-regulated in the BPH6G plants, suggesting that the majority positively regulate rice immunity against BPH. These DEGs were enriched in cellular development, cell wall organization, cellular component movement and hormone transport (Fig. 5c), which were consistent with the function of *BPH6*, which promotes exocytosis, participates in cell wall maintenance and reinforcement, and activates hormone signaling after BPH feeding [11]. In addition, the up-regulated DEGs were involved in the primary and secondary metabolite processes, suggesting that these metabolites play important roles in rice defense against BPH. Finally, 24 genes were selected as potential candidates for BPH resistance (Table 2). Most of the genes, excluding *LOC_Os02g45420* and *LOC_Os01g72270*, were highly upregulated (FC > 5) during the early stages in the BPH6G plants and highly downregulated during the late stages in WT, indicating their important roles in BPH response. The germin-like protein (GLP) gene family confers broad-spectrum resistance to pathogens and insects in plants through H_2_O_2_ production due to superoxide dismutase activity at the infection site [36, 37]. The overexpression of LTPs increases the resistance to pathogens and environmental stresses due to the hydrophobic protective layers of surface polymers [38]. Pectinesterase plays a regulatory role in mechanical stability and elongation of the cell wall in response to pathogen invasion in *Arabidopsis* [39]. Fasciclin-like arabinogalactan-proteins are implicated in plant growth and development, cell wall remodeling, hormone signaling modulation and pathogen defenses [40]. In addition, NB-ARC proteins, MYB transcription factors, ethylene response factors and HSP20 are all involved in pathogen resistance [41–44].

Integrated miRNA and mRNA expression analysis can help identify the functional miRNA-mRNA pairs related to host-insect interaction. In this study, 70 specific DEMs in the BPH6G plant (Fig. 2b) and 29 oppositely expressed miRNAs (Fig. 3b) corresponding to 656 target genes were detected under BPH attacking (Additional file 10: Table S6). However, only 34 miRNAs corresponding to 42 target genes might be potentially related to BPH response (Table 3). For example, the members of miR166 family, reported to positively regulate rice immunity against the blast fungus [26], were up-regulated in the BPH6G plants after BPH feeding (Fig. 3b). However, the targets of miR166 exhibited similar trend of expression in the BPH6G and WT plants after BPH feeding (Additional file 10: Table S6). Therefore, miR166 and its targets were excluded as BPH-related candidates. This phenomenon can be explained by the following: (1) most targets had the same expression trends in the BPH6G and WT plants after BPH feeding, (2) plants defense responses to insects include both systematic and local responses, and many targets may not be expressed at this point and (3) the accepted criteria for the DEMs and DEGs may miss key interactions. After integrated analysis of the DEMs and DEGs, several important miRNA-mRNA pairs involved in BPH stress were identified. miR156b-3p targeted to GDSL-like lipase in response to BPH (Table 3, Fig. 6). Previous studies have shown that miR156 silencing confers enhanced resistance to BPH [30], and GDSL lipases modulate immunity through lipid homeostasis [45]. Therefore, miR156b-3p may negatively regulate BPH resistance by targeting GDSL lipases. Members of the miR169 family, including miR169g/h/i-5p.1/i-5p.2/j/k/l/m/o, target some leucin rich repeat family proteins that play key roles in pattern recognition and the initiation of downstream responses [46]. In addition, members of miR1861 family target auxin response factors, miR396c-3p targets abscisic acid 8′-hydroxylase gene, and miR5513 targets ethylene-responsive transcription factor, suggesting that auxin, ABA and ethylene might all involved in the BPH response.

## Conclusion

In this study, 18 libraries were constructed for the BPH6G and WT genotypes before and after BPH feeding. These libraries were amplified and sequenced, and miRNAs and mRNAs related to BPH resistance were identified. We identified members of miR160, miR166, miR169, miR1861, miR319 and miR390 families, and other miRNAs that played important roles in the *BPH6*-mediated resistance to BPH. DEGs potentially involved in BPH responses included genes related to metabolic process, cellular development, cell wall organization, cellular component movement and hormone transport. Additionally, 34 miRNAs corresponding to 42 target genes were identified as candidates for BPH resistance miRNA-mRNA pairs. The integrated analyses of miRNAs and genes related to BPH resistance in rice provide the basis for further research probing the functions of miRNA and targets in the BPH response, and establish a molecular basis for further studies on how plants respond to BPH infestation.

## Methods

### Plant and insect materials

A 7.8 kb DNA fragment containing the *BPH6* gene with its native promoter, was amplified from Swarnalata (IRRI Acc. No. 33964), digested with *Kpn*I and inserted into the binary vector pCAMBIA1300, transformed into the susceptible wild type (WT) Nipponbare (IRRI Acc. No. 136196) through Agrobacterium-mediated method, and identified by Zhang et al. [47]. A voucher specimen of the *BPH6*-transgenic line has been deposited in the China Center for Type Culture Collection (No. P201907). Seeds were grown in plastic cups (9 cm in diameter and 15 cm in height) with 15 plants per cup, and maintained in a greenhouse with cycles of 32 ± 2 °C/14 h light and 26 ± 2 °C/10 h dark periods.

The BPH population in this study was were kept in the laboratory and maintained on 1-month-old plants of the susceptible rice cv Taichung Native1 (IRRI Acc. No. 00105) under controlled environmental conditions as described above in Wuhan University [48].

### Evaluation of rice resistance to BPH

At the four-leaf stage, the BPH6G and WT plants were infested with 8 s-instar BPH nymphs per seedling, and checked each day until all seedlings of WT died. Evaluations were carried out with three biological repeats for each line.

Host choice test was carried out as described [11], WT and BPH6G plants were grown diagonally in each bucket. At the four-leaf stage, twenty BPH nymphs at the third-instar stage were release in the buckets, and the number of nymphs settled per plant were counted at 6, 12, 24, 36, 48, 60 and 72 h after release. Ten buckets for each line were analyzed.

In BPH weight gain analysis, newly emerged brachypterous females, Parafilm sachets, 1-month-old WT and BPH6G lines were used as described by Shangguan et al. [48]. Weight increase relative to the initial weight were calculated BPH weight gain ratios. Experiments were performed five times with 10 replicates for each line.

### Sample collection

The endpoint method was used to collect samples through BPH treatment [29]. Although all processes began at different time, they were stopped at the same specified time. Seedlings were infested with 8 s-instar BPH per seedling at the four-leaf stage after 0, 6, 12, 24, 48, 60, and 72 h. For analysis, three biological replicates per treatment with 15 seedlings per replicate were used. Leaf sheaths were mixed for non-infested controls (0 h), infested early (6, 12 and 24 h) and infested late (48, 60 and 72 h). Samples were referred to as R0, R_early, and R_late for the BPH6G lines, and S0, S_early, and S_late for WT. Leaf sheathes were cut and frozen in liquid nitrogen, and stored at − 80 °C until use.

### RNA extraction

Infested and mock leaf sheathes were used for total RNA extraction using commercial RNAiso Plus kits (TaKaRa, code no. 9109). Concentrations of RNA were checked using Qubit fluorometric quantitation (Thermo Fisher Scientific, Wilmington, DE, USA), and integrity was verified on a Bio-Analyzer 2200 (Agilent Technologies, Waldbronn, Germany).

### Construction of the cDNA library and RNA mapping

cDNA library for each sample was constructed using NEBNext® Ultra™ directional RNA library prep kits (NEB, code no. E7420S), and quantified on a 150 bp paired-end run by Agilent2200 and sequenced by HiSeq X (Illumina, San Diego, CA). Clean reads were obtained after the removal of adaptors, low quality reads and reads with > 5% unknown nucleotides, and mapped on rice genome (TIGR7) using the Hisat2 [49]. Gene counts were obtained by HTseq and gene expression was determined using the RPKM method [50].

### miRNA library construction, sequencing and mapping

miRNA libraries were prepared using Ion Total RNA-Seq Kit v2.0 (Thermo Fisher, code no. 4475936). miRNA for construction were selected according to size by polyacrylamide gel electrophoresis and processed for Proton Sequencing as per commercially available protocols. A total of 18 small RNA libraries were constructed with the BPH6G plants (R) and WT (S) infested by BPH for non-infested (R0, S0), early feeding stages (R_early, S_early) and late feeding stages (R_late, S_late).

After deep sequencing, the raw data were evaluated in FAST-QC software (http://www.bioinformatics.babraham.ac.uk/projects/fastqc/), including the quality distribution of nucleotides, position specific sequencing quality, GC content, proportion of PCR duplication and k-mer frequency. Raw data were processed to remove low-quality reads, adaptor sequences, contaminant reads, and reads of < 20 nt and > 24 nt. All of the sequences were aligned in the NCBI GenBank (release 227.0) and Rfam (release 13.0) database, and mapped to the rice genome to identify and remove rRNA, tRNA, scRNA, snoRNA, snRNA and small RNAs mapped to exons or introns and repeat sequences (Additional file 2: Fig. S1B).

### Differential expression analysis of miRNAs and genes

Differentially expressed miRNAs and genes were filtered by EB-Seq algorithm after significance. *P*-values and FDR analyses were performed at absolute values of log_2_FC ≥ 1, *P* < 0.05, FDR < 0.05 [51].

### Target analysis

The psRNA target software (http://plantgrn.noble.org/psRNATarget/) was used to predict miRNA targets on mRNAs based on the default parameters.

### Analysis of GO (gene ontology) and KEGG pathway

GO annotations from NCBI (http://www.ncbi.nlm.nih.gov/) and GO (http://www.geneontology.org/) were downloaded. To identify DEGs pathways, the KEGG database was used. To identify significant GO and pathway categories, Fisher’s exact tests were applied under absolute values of *P* < 0.05 and FDR < 0.05 [52].

### qPCR analysis of miRNAs and mRNAs

For first strand cDNA synthesis, 2 μg total RNA were extracted using PrimeScript™ RT reagent Kits accompanied with gDNA Eraser (TaKaRa, code no. RR047A) and miRNAs were extracted using miRcute Plus miRNA First-Strand cDNA Kits (TIANGEN, code no. KR211). miRNAs were quantified by stem-loop RT-PCR [32]. Gene expression was analyzed by qPCR using SYBR green supermixes from Bio-Rad and CFX96 real-time system. Each experiment was performed in three biological replicates. The expression of miRNAs and genes were calculated through the 2^-ΔΔC (t)^ method [53] with internal reference genes *TBP* and U6, respectively. Primers are listed in Additional file 11: Table S7. One-way ANOVA was used for statistical analyses in Microsoft Excel.

### Validation of the predicted target genes of miRNAs

The role of miRNAs on the targets were investigated through counting the fluorescent cells [29]. One plasmid encoded pri-miRNA (miR156b-3p, miR169i-5p.2) was amplified from WT DNA and cloned into the binary vector pCXUN. The other containing the targets (*GDSL*, *LRR*) were amplified from WT cDNA and cloned into the binary vector pCXUN with YFP genes and HA tags. Constructs expressing miRNAs and the targets were transiently co-transfected into rice protoplasts isolated from 10-day-old WT stems. The fluorescent cells were imaged and numbered using a confocal microscope (FV10-ASW, Olympus). Protein expression was determined by Western blotting. Primers used in the experiments are listed in Additional file 11: Table S7.

## Supplementary information

**Additional file 1: Table S1.** Summary of small RNA sequences. Total Reads: raw data after sequencing. Clean Reads: reads after the removal of adaptors, low-quality reads, and reads of < 20 nt and > 24 nt. Mapped Reads: clean reads mapped on the miRbase. S: WT; R: the BPH6G plants; 0, non-infested; early: early feeding stage; late: late feeding stage.

**Additional file 2: Figure S1.** Size distribution and miRNAs annotation of the BPH6G and WT plants at non-infested, early and late feeding stages. **A** Length distribution of total reads. **B** Proportions of different kinds of small RNAs.

**Additional file 3: Table S2.** DEMs in both varieties before and after BPH feeding.

**Additional file 4: Table S3.** DEMs at the early and late feeding stages of the two varieties.

**Additional file 5: Table S4.** Summary of mRNA expression libraries. Total Reads: raw data after sequencing. Clean Reads: reads after the removal of adaptors, low quality tags, and single-copy tags. Mapped Reads: clean reads mapped on the rice genome. S: WT; R: the BPH6G plants; 0, non-infested; early: early feeding stages; late: late feeding stages.

**Additional file 6: Figure S2.** Hierarchical clustering analysis of DEGs of the BPH6G and WT plants after BPH feeding based on the log ratio of FPKM data. Red and green indicate upregulated and downregulated DEGs, respectively. Each row shows genes and each column represents a comparison.

**Additional file 7: Figure S3.** Venn diagrams of the number of upregulated and downregulated DEGs (**A**), and opposite expression DEGs (**B**) of the BPH6G and WT plants at different feeding stages.

**Additional file 8: Figure S4.** GO (Gene Ontology) analysis. Biological process, cellular component, and molecular function of up-(**A**) and down-regulated (**B**) DEGs in R_early/R0 and R_late/R0 respectively, and up- (**C**) and down-regulated (**D**) DEGs in S_early/S0 and S_late/S0 respectively (*P* < 0.05). The x-axis and y-axis indicate names of clusters and genes in a category, respectively.

**Additional file 9: Table S5.** KEGG pathway enrichment analysis of DEGs appeared opposite expression at early or late feeding stages of two varieties.

**Additional file 10: Table S6.** Integrated analysis of BPH resistance related miRNAs and their target genes.

**Additional file 11: Table S7.** Primer sequences for qRT-PCR and the transformation of rice protoplasts.

## Data Availability

The raw sequence data of small RNA and transcriptome during this study could be found in the National Center for Biotechnology Information (NCBI) under the accession number GSE123148. This public accession is currently private and is scheduled to be released on Dec 31, 2019. I have received administrative permission to access and use these. https://www.ncbi.nlm.nih.gov/geo/query/acc.cgi?acc=GSE123148.

## Abbreviations

AOS2: Allene oxide synthase 2
BPH: Brown planthopper
DEG: Differentially expressed gene
DEM: Differentially expressed microRNA
FPKM: Fragments per kilobase of exon per million fragments
IPT10: Isopentenyl-transferase 10
IRRI: International Rice Research Institute
MiRNA: MicroRNA
PAL: Phenylalanine ammonia-lyase
ScRNA: Small cytoplasmic RNA
SnoRNA: Small nucleolar RNA
SnRNA: Small nuclear RNA

## References

[1] Cheng X, Zhu L, He G (2013). Towards understanding of molecular interactions between rice and the brown planthopper. Mol Plant.

[2] Jing S, Zhao Y, Du B, Chen R, Zhu L, He G (2017). Genomics of interaction between the brown planthopper and rice. Curr Opin Insect Sci.

[3] Pathak MD, Cheng CH, Fortuno ME (1969). Resistance to Nephotettix impicticeps and Nilaparvata lugens in varieties of rice. Nature.

[4] Du B, Zhang W, Liu B, Hu J, Wei Z, Shi Z, He R, Zhu L, Chen R, Han B (2009). Identification and characterization of Bph14, a gene conferring resistance to brown plant hopper in rice. Proc Natl Acad Sci U S A.

[5] Tamura Y, Hattori M, Yoshioka H, Yoshioka M, Takahashi A, Wu J, Sentoku N, Yasui H (2014). Map-based cloning and characterization of a brown planthopper resistance gene BPH26 from *Oryza sativa* L. ssp indica cultivar ADR52. Sci Rep.

[6] Liu Y, Wu H, Chen H, Liu Y, He J, Kang H, Sun Z, Pan G, Wang Q, Hu J (2015). A gene cluster encoding lectin receptor kinases confers broad-spectrum and durable insect resistance in rice. Nat Biotechnol.

[7] Wang Y, Cao L, Zhang Y, Cao C, Liu F, Huang F, Qiu Y, Lou X (2015). Map-based cloning and characterization of BPH29, a B3 domain-containing recessive gene conferring brown planthopper resistance in rice. J Exp Bot.

[8] Ren J, Gao F, Wu X, Lu X, Zeng L, Lv J, Su X, Lou H, Ren G (2016). Bph32, a novel gene encoding an unknown SCR domaincontaining protein, confers resistance against the brown planthopper in rice. Sci Rep.

[9] Ji H, Kim SR, Kim YH, Suh JP, Park HM, Sreenivasulu N, Misra G, Kim SM, Hechanova SL, Kim H (2016). Map-based cloning and characterization of the BPH18 gene from wild rice conferring resistance to brown planthopper (BPH) insect pest. Sci Rep.

[10] Zhao Y, Huang J, Wang Z, Jing S, Wang Y, Ouyang Y, Cai B, Xin XF, Liu X, Zhang C (2016). Allelic diversity in an NLR gene BPH9 enables rice to combat planthopper variation. Proc Natl Acad Sci U S A.

[11] Guo J, Xu C, Wu D, Zhao Y, Qiu Y, Wang X, Ouyang Y, Cai B, Liu X, Jing S (2018). *Bph6* encodes an exocyst**-**localized protein and confers broad resistance to planthoppers in rice. Nat Genet.

[12] Zhang F, Zhu L, He G (2004). Differential gene expression in response to brown planthopper feeding in rice. J Plant Physiol.

[13] Yuan H, Chen X, Zhu L, He G (2005). Identification of genes responsive to brown planthopper *Nilaparvata lugens* Stål (Homoptera: Delphacidae) feeding in rice. Planta.

[14] Wang Y, Wang X, Yuan H, Chen R, Zhu L, He R, He G (2008). Responses of two contrasting genotypes of rice to brown planthopper. Mol Plant-Microbe Interact.

[15] Wang Y, Guo H, Li H, Zhang H, Miao X (2012). Identification of transcription factors potential related to brown planthopper resistance in rice via microarray expression profiling. BMC Genomics.

[16] Li C, Luo C, Zhou Z, Wang R, Ling F, Xiao L, Lin Y, Chen H (2017). Gene expression and plant hormone levels in two contrasting rice genotypes responding to brown planthopper infestation. BMC Plant Biol.

[17] Lv W, Du B, Shangguan X, Zhao Y, Pan Y, Zhu L, He Y, He G (2014). BAC and RNA sequencing reveal the brown planthopper resistance gene *BPH15* in a recombination cold spot that mediates a unique defense mechanism. BMC Genomics.

[18] Axtell MJ, Meyers BC (2018). Revisiting criteria for plant MicroRNA annotation in the era of big data. Plant Cell.

[19] Bartel DP (2009). MicroRNAs: target recognition and regulatory functions. Cell.

[20] Marin E, Jouannet V, Herz A, Lokerse AS, Weijers D, Vaucheret H, Nussaume L, Crespi MD, Maizel A (2010). miR390, *Arabidopsis* TAS3 tasiRNAs, and their AUXIN RESPONSE FACTOR targets define an autoregulatory network quantitatively regulating lateral root growth. Plant Cell.

[21] Peng T, Sun H, Qiao M, Zhao Y, Du Y, Zhang J, Li J, Tang G, Zhao Q (2014). Differentially expressed microRNA cohorts in seed development may contribute to poor grain filling of inferior spikelets in rice. BMC Plant Biol.

[22] Magnrauthia SK, Bhogireddy S, Agarwal S, Prasanth VV, Voleti SR, Neelamaraju S, Subrahmanyam D (2017). Genome-wide changes in microRNA expression during short and prolonged heat stress and recovery in contrasting rice cultivars. J Exp Bot.

[23] Zhang S, Yue Y, Sheng L, Wu Y, Fan G, Li A, Hu X, Shangguan M, Wei C (2013). PASmiR: a literature-curated database for miRNA molecular regulation in plant response to abiotic stress. BMC Plant Biol.

[24] Natarajan B, Kalsi HS, Godbole P, Malankar N, Thiagarayaselvam A, Siddappa S, Thulasiram HV, Chakrabarti SK, Banerjee AK (2018). MiRNA160 is associated with local defense and systemic acquired resistance against *Phytophthora infestans* infection in potato. J Exp Bot.

[25] Li Y, Zhao SL, Li JL, Hu XH, Wang H, Cao XL, Xu XJ, Zhao ZX, Xiao ZY, Yang N (2017). Osa-miR169 negatively regulates rice immunity against the blast fungus *Magnaporthe oryzae*. Front Plant Sci.

[26] Salvador-Guirao R, Hsing YI, San SB (2018). The polycistronic miR166k-166h positively regulates rice immunity via post-transcriptional control of *EIN2*. Front Plant Sci.

[27] Zhang C, Ding Z, Wu K, Yang L, Li Y, Yang Z, Shi S, Liu X, Zhao S, Yang Z (2016). Suppression of jasmonic acid-mediated defense by viral-inducible microRNA319 facilitates virus infection in rice. Mol Plant.

[28] Zhang X, Bao Y, Shan D, Wang Z, Song X, Wang Z, Wang J, He L, Wu L, Zhang Z (2018). M*agnaporthe oryzae* induces the expression of a microRNA to suppress the immune response in rice. Plant Physiol.

[29] Wu Y, Lv W, Hu L, Rao W, Zeng Y, Zhu L, He Y, He G (2017). Identification and analysis of brown planthopper-responsive microRNAs in resistant and susceptible rice plants. Sci Rep.

[30] Ge Y, Han J, Zhou G, Xu Y, Ding Y, Shi M, Guo C, Wu G (2018). Silencing of miR156 confers enhanced resistance to brown planthopper in rice. Planta.

[31] Dai Z, Tan J, Zhou C, Yang X, Yang F, Zhang S, Sun S, Miao X, Shi Z (2019). The OsmiR396-OsGRF8-OsF3H-flavonoid pathway mediates resistance to the brown planthopper in rice (*Oryza sativa*). Plant Biotechnol J.

[32] Chen C, Ridzon DA, Broomer AJ, Zhou Z, Lee DH, Nguyen JT, Barbisin M, Xu NL, Mahuvakar VR, Andersen MR (2005). Real-time quantification of microRNAs by stem-loop RT-PCR. Nucleic Acids Res.

[33] Hu J, Zhou J, Peng X, Xu H, Liu C, Du B, Yuan H, Zhu L, He G (2011). The *Bphi008a* gene interacts with the ethylene pathway and transcriptionally regulates *MAPK* genes in the response of rice to brown planthopper feeding. Plant Physiol.

[34] Dai X, Zhuang Z, Zhao PX (2018). psRNATarget: a plant small RNA target analysis server (2017 release). Nucleic Acids Res.

[35] Sattar S, Tompson GA (2016). Small RNA regulators of plant-hemipteran interactions: micromanagers with versatile roles. Front Plant Sci.

[36] Manosalva PM, Davison RM, Liu B, Zhu X, Hulbert SH, Leung H, Leach JE (2009). A germin-like protein gene family functions as a complex quantitative trait locus conferring broad-spectrum disease resistance in rice. Plant Physiol.

[37] Liu Q, Yang J, Yan S, Zhang S, Zhao J, Wang W, Yang T, Wang X, Mao X, Dong J (2016). The germin-like protein OsGLP2-1 enhances resistance to fungal blast and bacterial blight in rice. Plant Mol Biol.

[38] Jung HW, Kim KD, Hwang BK (2005). Identification of pathogen-responsive regions in the promoter of a pepper lipid transfer protein gene (CALTPI) and the enhanced resistance of the CALTPI transgenic Arabidopsis against pathogen and environmental stresses. Planta.

[39] Bethke G, Grundman RE, Sreekanta S, Truman W, Katagiri F, Glazebrook J (2014). Arabidopsis PECTIN METHYLESTERASEs contribute to immunity against *Pseudomonas syringae*. Plant Physiol.

[40] Tan L, Showalter AM, Egelund J, Hernandez-Sanchez A, Doblin MS, Bacic A (2012). Arabinogalactan-proteins and the research challenges for these enigmatic plant cell surface proteoglycans. Front Plant Sci.

[41] Shirasu K (2009). The HSP90-SGT1 chaperone complex for NLR immune sensors. Annu Rev Plant Biol.

[42] Liu D, Chen X, Liu J, Ye J, Guo Z (2012). The rice ERF transcription factor OsERF922 negatively regulates resistance to *Magnaporthe oryzae* and salt tolerance. J Exp Bot.

[43] Jacob F, Vernaldi S, Maekawa T (2013). Evolution and conservation of plant NLR functions. Front Immunol.

[44] Li W, Zhu Z, Chern M, Yin J, Yang C, Ran L, Cheng M, He M, Wang K, Wang J (2017). A natural allele of a transcription factor in rice confers broad-spectrum blast resistance. Cell.

[45] Gao M, Yin X, Yang W, Lam SM, Tong X, Liu J, Wang X, Li Q, Shui G, He Z (2017). GDSL lipases modulate immunity through lipid homeostasis in rice. PLoS Pathog.

[46] Macho AP, Zipfel C (2014). Plant PRRs and the activation of innate immune signaling. Mol Cell.

[47] Zhang J, Guan W, Huang C, Hu Y, Chen Y, Guo J, Zhou C, Chen R, Du B, Zhu L (2019). Combining next-generation sequencing and single-molecule sequencing to explore brown plant hopper responses to contrasting genotypes of japonica rice. BMC Genomics.

[48] Shangguan X, Zhang J, Liu B, Zhao Y, Wang H, Wang Z, Guo J, Rao W, Jing S, Guan W (2018). A mucin-like protein of planthopper is required for feeding and induces immunity response in plants. Plant Physiol.

[49] Kim D, Langmead B, Salzberg SL (2015). HISAT: a fast spliced aligner with low memory requirements. Nat Methods.

[50] Anders S, Pyl PT, Huber W (2015). HTSeq—A Python framework to work with high-throughput sequencing data. Bioinformatics.

[51] Leng N, Dawson JA, Thomson JA, Ruotti V, Rissman AI, Smits BM, Haag JD, Gould MN, Stevart RM, Kendziorski C (2013). EBSeq: an empirical Bayes hierarchical model for inference in RNA-seq experiments. Bioinformatics.

[52] Draghici S, Khatri P, Tarca AL, Amin K, Done A, Voichita C, Georgescu C, Romero R (2007). A systems biology approach for pathway level analysis. Genome Res.

[53] Schmittgen TD, Livak KJ (2008). Analyzing real-time PCR data by the comparative C_(T)_ method. Nat Protoc.

