# Supplementary material for: A combined microRNA and transcriptome analyses illuminates the resistance response of rice against brown planthopper
Source: BMC Genomics. 2020 Feb 10;21:144. doi: 10.1186/s12864-020-6556-6 (PMC7011362; doi:10.1186/s12864-020-6556-6)
Supplement: Supplementary file 1 — Additional file 1: Table S1. Summary of small RNA sequences. Total Reads: raw data after sequencing. Clean Reads: reads after the removal of adaptors, low-quality reads, and reads of < 20 nt and > 24 nt. Mapped Reads: clean reads mapped on the miRbase. S: WT; R: the BPH6G plants; 0, non-infested; early: early feeding stage; late: late feeding stage. [file 12864_2020_6556_MOESM1_ESM.docx]

**Table S1** **Summary of small RNA sequences data**.

| Samples | Total reads | Clean reads | Mapped reads on miRBase | Mapped rate (%) |
| --- | --- | --- | --- | --- |
| S0_1 | 10,765,888 | 6,507,818 | 1,502,741 | 23.09 |
| S0_2 | 13,036,033 | 7,140,298 | 1,439,346 | 20.16 |
| S0_3 | 12,326,311 | 8,291,591 | 1,761,820 | 21.25 |
| S_early_1 | 11,149,266 | 4,743,399 | 1,257,950 | 26.35 |
| S_early_2 | 9,506,071 | 4,503,508 | 1,066,585 | 23.68 |
| S_early_3 | 10,468,810 | 4,875,946 | 1,165,480 | 23.90 |
| S_late_1 | 11,865,761 | 5,488,219 | 1,126,154 | 20.52 |
| S_late_2 | 10,146,511 | 5,052,571 | 1,004,235 | 19.88 |
| S_late_3 | 12,854,519 | 5,914,336 | 1,442,464 | 24.39 |
| R_0_1 | 11,983,131 | 7,623,012 | 2,337,445 | 30.66 |
| R_0_2 | 9,416,313 | 6,130,761 | 1,902,795 | 31.04 |
| R_0_3 | 12,417,777 | 8,119,955 | 2,503,406 | 30.83 |
| R_early_1 | 9,034,925 | 4,837,652 | 1,721,135 | 35.58 |
| R_early_2 | 12,520,216 | 4,778,110 | 1,529,498 | 32.01 |
| R_early_3 | 13,709,368 | 5,215,770 | 1,636,092 | 31.37 |
| R_late_1 | 10,275,771 | 5,789,347 | 1,370,518 | 23.67 |
| R_late_2 | 9,289,358 | 5,639,456 | 1,329,172 | 23.57 |
| R_late_3 | 14,016,694 | 8,547,717 | 2,467,766 | 28.87 |

Note:

Total Reads: the raw data after sequencing

Clean Reads: the reads after filtering out all the low-quality reads, poly A, incorrect adaptors and reads of < 18 nt

Mapped Reads: the reads of the clean reads that could be mapped to the miRbase

S: Nipponbare; R: the *BPH6*-transgenic plants; 0, non-infested; early: early feeding stage; late: late feeding stage.
