# Supplementary figures and images for: A combined microRNA and transcriptome analyses illuminates the resistance response of rice against brown planthopper
Source: BMC Genomics. 2020 Feb 10;21:144. doi: 10.1186/s12864-020-6556-6 (PMC7011362; doi:10.1186/s12864-020-6556-6)

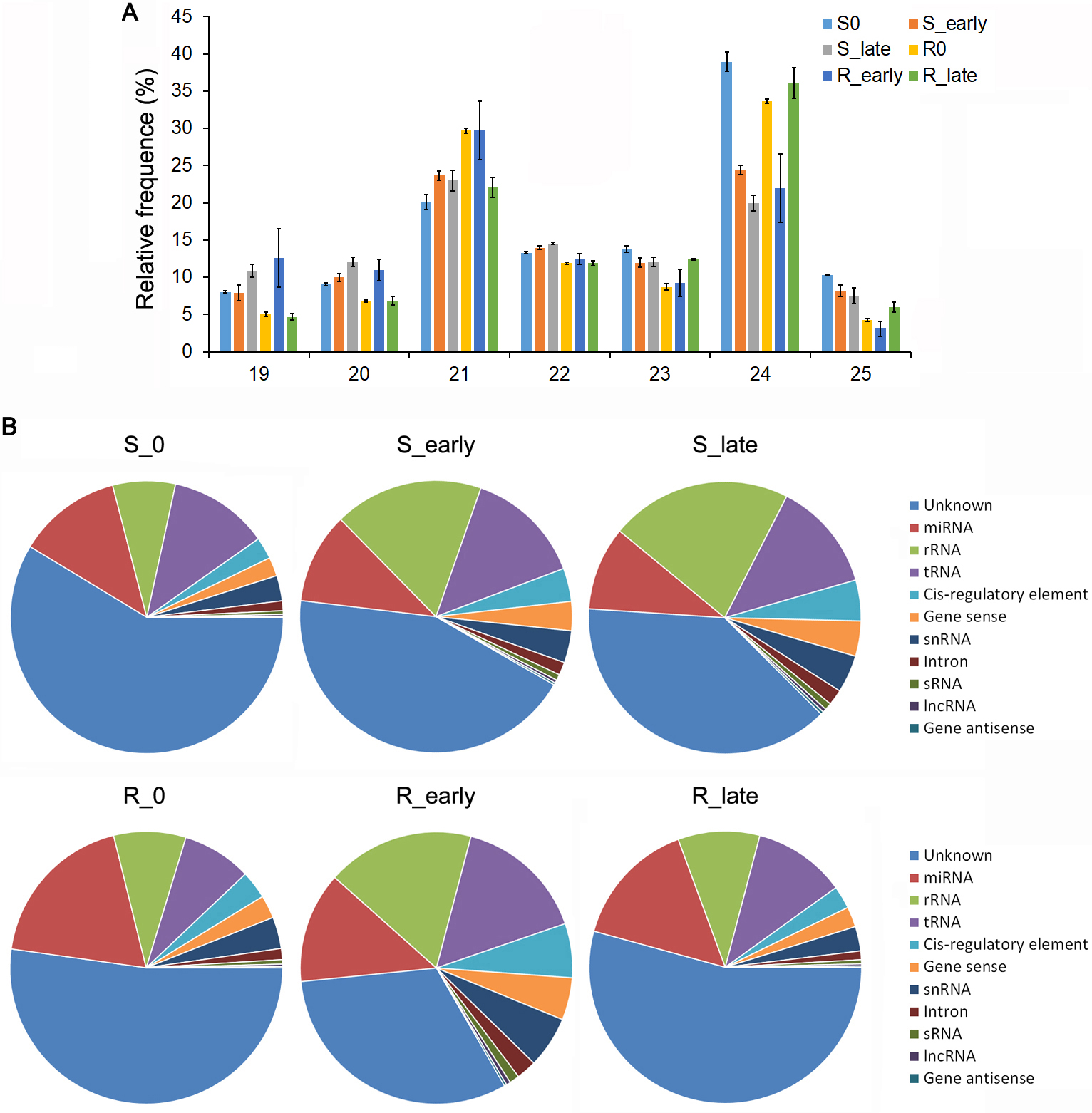

Supplement: Supplementary file 2 — Additional file 2: Figure S1. Size distribution and miRNAs annotation of the BPH6G and WT plants at non-infested, early and late feeding stages. A Length distribution of total reads. B Proportions of different kinds of small RNAs. [file 12864_2020_6556_MOESM2_ESM.jpg]

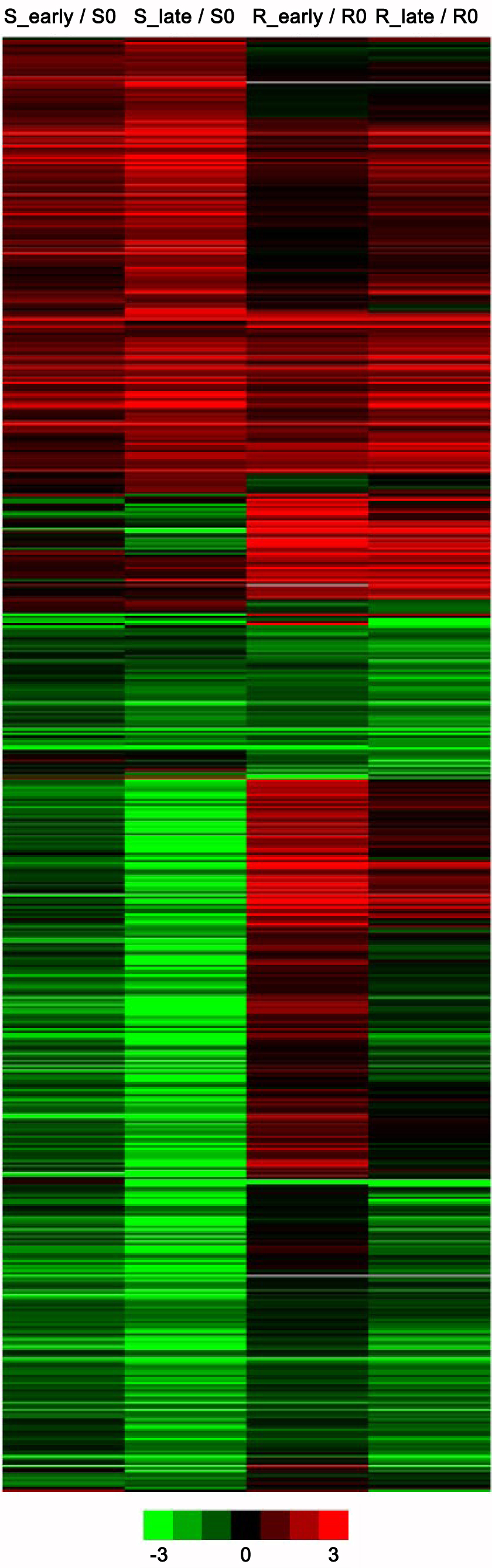

Supplement: Supplementary file 6 — Additional file 6: Figure S2. Hierarchical clustering analysis of DEGs of the BPH6G and WT plants after BPH feeding based on the log ratio of FPKM data. Red and green indicate upregulated and downregulated DEGs, respectively. Each row shows genes and each column represents a comparison. [file 12864_2020_6556_MOESM6_ESM.jpg]

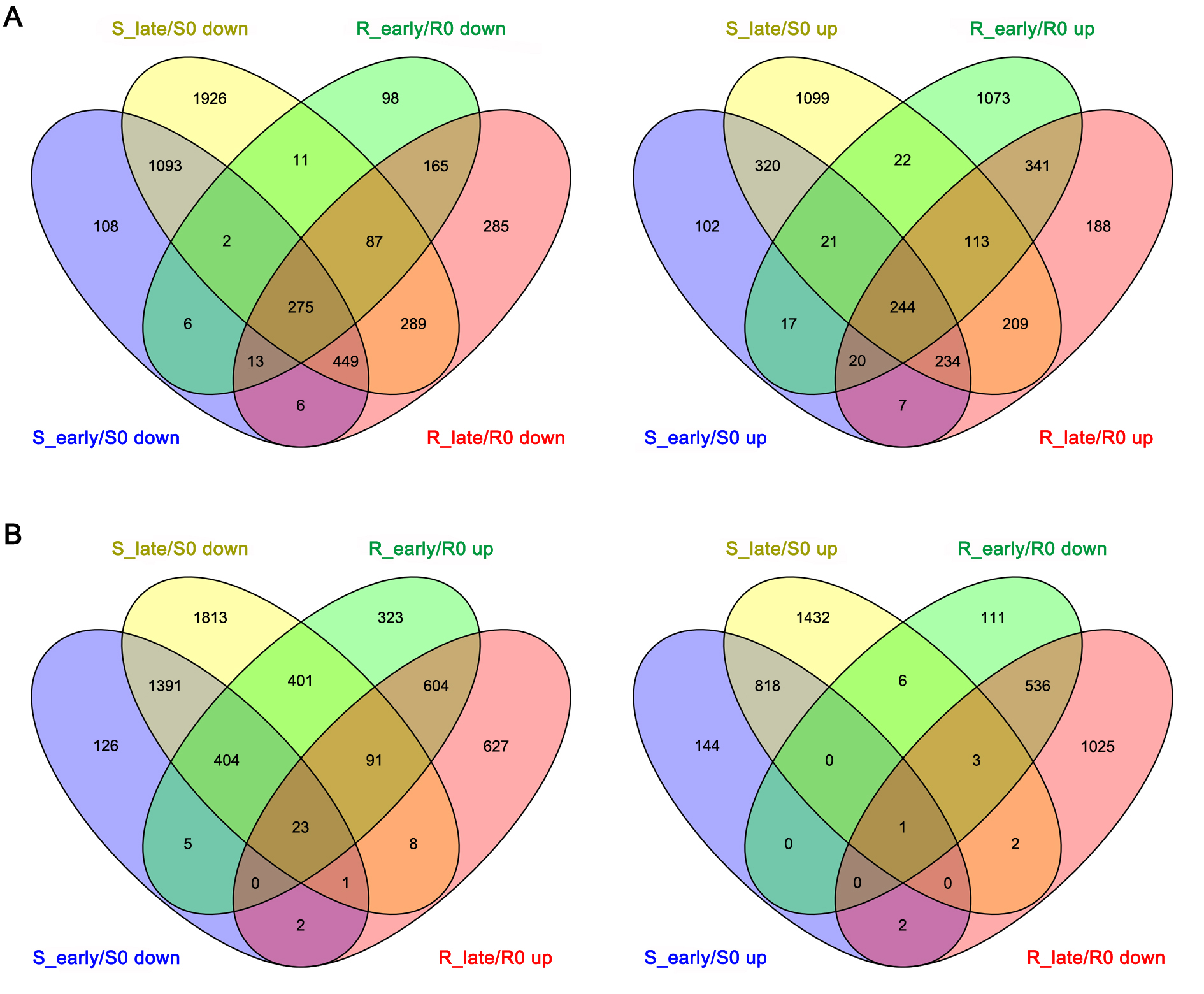

Supplement: Supplementary file 7 — Additional file 7: Figure S3. Venn diagrams of the number of upregulated and downregulated DEGs (A), and opposite expression DEGs (B) of the BPH6G and WT plants at different feeding stages. [file 12864_2020_6556_MOESM7_ESM.jpg]

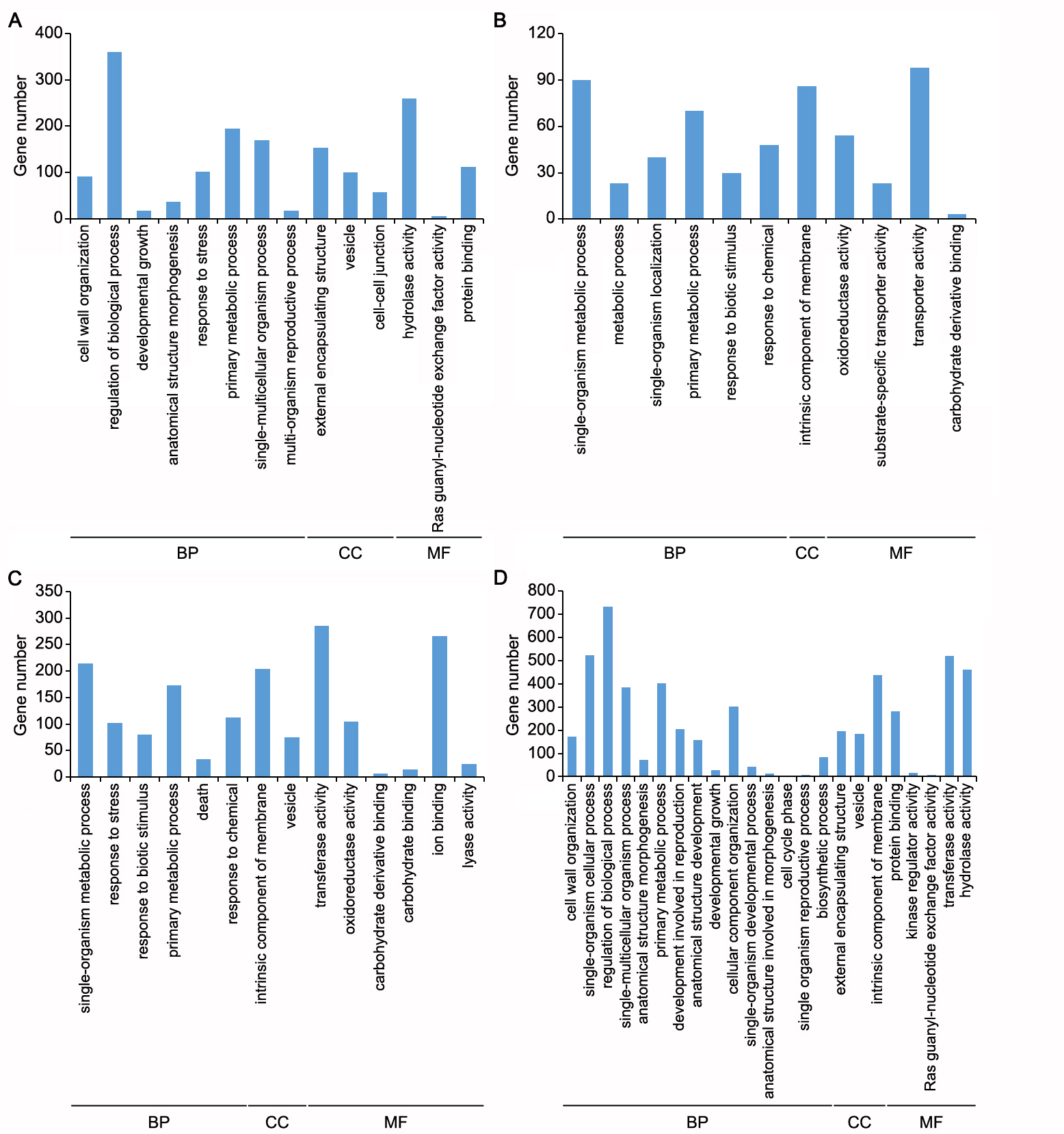

Supplement: Supplementary file 8 — Additional file 8: Figure S4. GO (Gene Ontology) analysis. Biological process, cellular component, and molecular function of up-(A) and down-regulated (B) DEGs in R_early/R0 and R_late/R0 respectively, and up- (C) and down-regulated (D) DEGs in S_early/S0 and S_late/S0 respectively (P < 0.05). The x-axis and y-axis indicate names of clusters and genes in a category, respectively. [file 12864_2020_6556_MOESM8_ESM.jpg]
