# Supplementary material for: A combined microRNA and transcriptome analyses illuminates the resistance response of rice against brown planthopper
Source: BMC Genomics. 2020 Feb 10;21:144. doi: 10.1186/s12864-020-6556-6 (PMC7011362; doi:10.1186/s12864-020-6556-6)
Supplement: Supplementary file 5 — Additional file 5: Table S4. Summary of mRNA expression libraries. Total Reads: raw data after sequencing. Clean Reads: reads after the removal of adaptors, low quality tags, and single-copy tags. Mapped Reads: clean reads mapped on the rice genome. S: WT; R: the BPH6G plants; 0, non-infested; early: early feeding stages; late: late feeding stages. [file 12864_2020_6556_MOESM5_ESM.docx]

**Table S4 Summary of mRNA expression libraries**

| Samples | Total reads | Clean reads | Mapped reads | No. of mapped genes |
| --- | --- | --- | --- | --- |
| S0_1 | 95,904,402 | 91,490,042  (95.40%) | 83,067,269  (90.79%) | 29,829 |
| S0_2 | 107,440,012 | 102,911,942  (95.79%) | 93,484,366  (90.84%) | 30,006 |
| S0_3 | 98,900,992 | 93,776,916  (94.82%) | 85,012,537  (90.65%) | 29,806 |
| S_early_1 | 95,975,518 | 91,468,778  (95.30%) | 80,749,424  (88.28%) | 29,505 |
| S_early_2 | 111,697,630 | 105,819,738  (94.74%) | 92,950,415  (87.84%) | 29,569 |
| S_early_3 | 100,272,166 | 95,154,042  (94.90%) | 82,017,416  (86.19%) | 29,435 |
| S_late_1 | 104,064,522 | 98,948,810  (95.08%) | 81,797,681  (82.67%) | 28,838 |
| S_late_2 | 110,801,772 | 105,324,228  (95.06%) | 87,905,786  (83.46%) | 29,023 |
| S_late_3 | 104,077,688 | 98,346,596  (94.49%) | 81,728,726  (83.10%) | 28,874 |
| R_0_1 | 95,834,398 | 91,627,526  (95.61%) | 81,914,360  (89.40%) | 29,028 |
| R_0_2 | 108,010,044 | 103,354,580  (95.69%) | 92,297,408  (89.30%) | 29,232 |
| R_0_3 | 110,390,690 | 105,687,764  (95.74%) | 95,107,581  (89.99%) | 29,128 |
| R_early_1 | 108,706,076 | 103,876,388  (95.56%) | 91,109,432  (87.71%) | 30,094 |
| R_early_2 | 95,471,364 | 91,310,560  (95.64%) | 80,661,552  (88.34%) | 30,383 |
| R_early_3 | 104,334,822 | 100,142,084  (95.98%) | 87,121,926  (87.00%) | 30,012 |
| R_late_1 | 110,627,486 | 104,951,606  (94.85%) | 90,370,353  (86.12%) | 29,197 |
| R_late_2 | 95,908,290 | 90,714,672  (94.58%) | 77,010,151  (84.89%) | 29,056 |
| R_late_3 | 96,735,946 | 91,901,244  (95.00%) | 77,840,558  (84.70%) | 28,988 |

Note:

Total Reads: the raw data after sequencing

Clean Reads: the reads after filtering out low-quality tags, unexpected-length tags, and single-copy tags

Mapped Reads: the reads of the clean reads that could be mapped to the rice genome
S: Nipponbare; R: Bph6-transgenic plants; 0, non-infested; early: early infested by BPH; late: late infested by BPH.
