# Supplementary material for: A combined microRNA and transcriptome analyses illuminates the resistance response of rice against brown planthopper
Source: BMC Genomics. 2020 Feb 10;21:144. doi: 10.1186/s12864-020-6556-6 (PMC7011362; doi:10.1186/s12864-020-6556-6)
Supplement: Supplementary file 9 — Additional file 9: Table S5. KEGG pathway enrichment analysis of DEGs appeared opposite expression at early or late feeding stages of two varieties. [file 12864_2020_6556_MOESM9_ESM.docx]

**Table S5** KEGG pathway enrichment analysis of differently expressed genes appeared opposite expression in early or late feeding stages of two rice genotypes.

|  | PathwayID | PathwayTerm | P-Value | Enrichment |
| --- | --- | --- | --- | --- |
| Up regulated | KO00903 | Limonene and pinene degradation | 0.0030 | 9.9414 |
|  | KO00040 | Pentose and glucuronate interconversions | 0.0061 | 7.7803 |
|  | KO00500 | Starch and sucrose metabolism | 0.0124 | 3.5087 |
|  | KO00945 | Stilbenoid, diarylheptanoid and gingerol biosynthesis | 0.0162 | 9.9414 |
|  | KO00280 | Valine, leucine and isoleucine degradation | 0.0245 | 4.7091 |
|  | KO04650 | Natural killer cell mediated cytotoxicity | 0.0283 | 7.4561 |
|  | KO00460 | Cyanoamino acid metabolism | 0.0354 | 6.6276 |
|  | KO00330 | Arginine and proline metabolism | 0.0359 | 4.0670 |
|  | KO00940 | Phenylpropanoid biosynthesis | 0.0402 | 3.8901 |
|  | KO00905 | Brassinosteroid biosynthesis | 0.0495 | 19.8829 |
| Down regulated | KO00520 | Amino sugar and nucleotide sugar metabolism | 0.0041 | 17.7269 |
|  | KO00904 | Diterpenoid biosynthesis | 0.0176 | 56.5897 |
